# QCScreen: a software tool for data quality control in LC-HRMS based metabolomics

**DOI:** 10.1186/s12859-015-0783-x

**Published:** 2015-10-24

**Authors:** Alexandra Maria Simader, Bernhard Kluger, Nora Katharina Nicole Neumann, Christoph Bueschl, Marc Lemmens, Gerald Lirk, Rudolf Krska, Rainer Schuhmacher

**Affiliations:** Center for Analytical Chemistry, Department of Agrobiotechnology (IFA-Tulln), University of Natural Resources and Life Sciences, Vienna (BOKU), Konrad Lorenz Strasse 20, 3430 Tulln, Austria; Institute for Biotechnology in Plant Production, Department of Agrobiotechnology (IFA-Tulln), University of Natural Resources and Life Sciences, Vienna (BOKU), Konrad Lorenz Strasse 20, 3430 Tulln, Austria; University of Applied Sciences Upper Austria, School of Informatics, Communications and Media, Softwarepark 11, 4232 Hagenberg, Austria

**Keywords:** QC, LC-HRMS, Retention time shift, Mass accuracy shift, mzXML

## Abstract

**Background:**

Metabolomics experiments often comprise large numbers of biological samples resulting in huge amounts of data. This data needs to be inspected for plausibility before data evaluation to detect putative sources of error e.g. retention time or mass accuracy shifts. Especially in liquid chromatography-high resolution mass spectrometry (LC-HRMS) based metabolomics research, proper quality control checks (e.g. for precision, signal drifts or offsets) are crucial prerequisites to achieve reliable and comparable results within and across experimental measurement sequences. Software tools can support this process.

**Results:**

The software tool QCScreen was developed to offer a quick and easy data quality check of LC-HRMS derived data. It allows a flexible investigation and comparison of basic quality-related parameters within user-defined target features and the possibility to automatically evaluate multiple sample types within or across different measurement sequences in a short time. It offers a user-friendly interface that allows an easy selection of processing steps and parameter settings. The generated results include a coloured overview plot of data quality across all analysed samples and targets and, in addition, detailed illustrations of the stability and precision of the chromatographic separation, the mass accuracy and the detector sensitivity. The use of QCScreen is demonstrated with experimental data from metabolomics experiments using selected standard compounds in pure solvent. The application of the software identified problematic features, samples and analytical parameters and suggested which data files or compounds required closer manual inspection.

**Conclusions:**

QCScreen is an open source software tool which provides a useful basis for assessing the suitability of LC-HRMS data prior to time consuming, detailed data processing and subsequent statistical analysis. It accepts the generic mzXML format and thus can be used with many different LC-HRMS platforms to process both multiple quality control sample types as well as experimental samples in one or more measurement sequences.

**Electronic supplementary material:**

The online version of this article (doi:10.1186/s12859-015-0783-x) contains supplementary material, which is available to authorized users.

## Background

Many metabolomics studies involve the measurement of large numbers of experimental samples, which makes it necessary to combine analytical data originating from different measurements, several sequences or sometimes even from different experiments.

The comprehensive evaluation of the generated measurement data is a laborious and time-consuming task. In case of liquid chromatography-high resolution mass spectrometry (LC-HRMS) detector sensitivity drifts, retention time or mass accuracy shifts within or across different measurement sequences do frequently occur in practice and can further complicate data evaluation. Software tools which enable a rapid check of data quality and consistency can greatly support the decision if a particular data set is suitable for further data processing and subsequent statistical analysis. Such tools are therefore of general interest in metabolomics. Stable isotopically labelled analytes [1–3] or labelled biological samples [4, 5] and various types of control samples are widely used for quality control (QC) [6, 7]. Internal standardisation can be regarded the gold standard for QC purposes as this technique enables “within” sample compensation of ion suppression and technical variability as for example reviewed in [8]. In contrast to that, QC samples such as solvent blanks, selected standard mixtures and representative pooled matrix samples can be used for “between sample” standardisation. Typically, QC samples are analysed periodically within and across measurement sequences to monitor analytical sensitivity, precision, stability or sample carry-over of the analytical process [9]. Based on the different (QC) sample categories, both multivariate [10–13] and univariate [9] methods have been described to evaluate and illustrate the analytical performance of individual samples and analytical features in the LC-HRMS data set(s) under investigation. Obviously, visual inspection of short- and long-term stability of critical parameters such as the MS signal abundance, retention time and mass accuracy constitutes the first and most straightforward step in quickly assessing data integrity. While some of the available comprehensive software programs for the processing of metabolomics data support different QC checks [7, 13–15], to the best of the authors knowledge no free, standalone software tool is available that provides a quick and intuitive assessment of quality in LC-HRMS raw data from several measurement sequences based on the inspection of basic quality-related parameters.

The presented software QCScreen is a novel, freely available software tool that allows the flexible investigation of selected feature parameters to assess data quality within and across LC-HRMS measurement sequences. QCScreen inspects LC-HRMS data for the presence and analytical behaviour of user - definable target features. Both graphical and tabular outputs of the different, evaluated quality parameters, which allow for a quick check, are automatically generated in support of both descriptive as well as prescriptive feature-based analytical quality assessment.

## Implementation

QCScreen can be used to detect and examine predefined analytical features from full scan LC-HRMS data with the aim to provide a quick and easy-interpretable graphical overview of data quality.

Data files referring to specified sample categories (e.g. blanks, QC samples, QC standards, matrix QCs or experimental samples) originating from one or multiple measurement sequences can be selected and inspected using predefined features. For the features and samples under investigation, QCScreen visualizes the stability and precision of the chromatographic separation step, MS sensitivity, *m/z* value and mass accuracy (±ppm) over time. The generated graphical illustrations and a coloured quality overview table enable quickly spotting putatively problematic data, i.e. features, samples or measurement sequences. QCScreen was implemented in the Python programming language (v. 2.6) and uses the Qt framework for the graphical user interface (GUI), R Version 2.12 and SQlite for data management.

QCScreen loads the centroided raw data in the mzXML format [16]. The GUI of the software tool contains a main area for selection of measurement sequences, sample type categories and features to be investigated and provides the option to define parameters, display settings as well as tolerance limits for the parameters. The then observed feature characteristics are evaluated against predefined or data-based performance criteria. For each feature, QCScreen creates an illustration of extracted ion chromatogram (EIC), retention time (tR), mass-to-charge ratio (*m/z*), mass accuracy and feature area across the provided data files. Finally, evaluation results of all tested features and parameters are saved in tabular form.

In the following the implemented sections are described.

### Sequence input and sample type selection

**Fig. 1:**
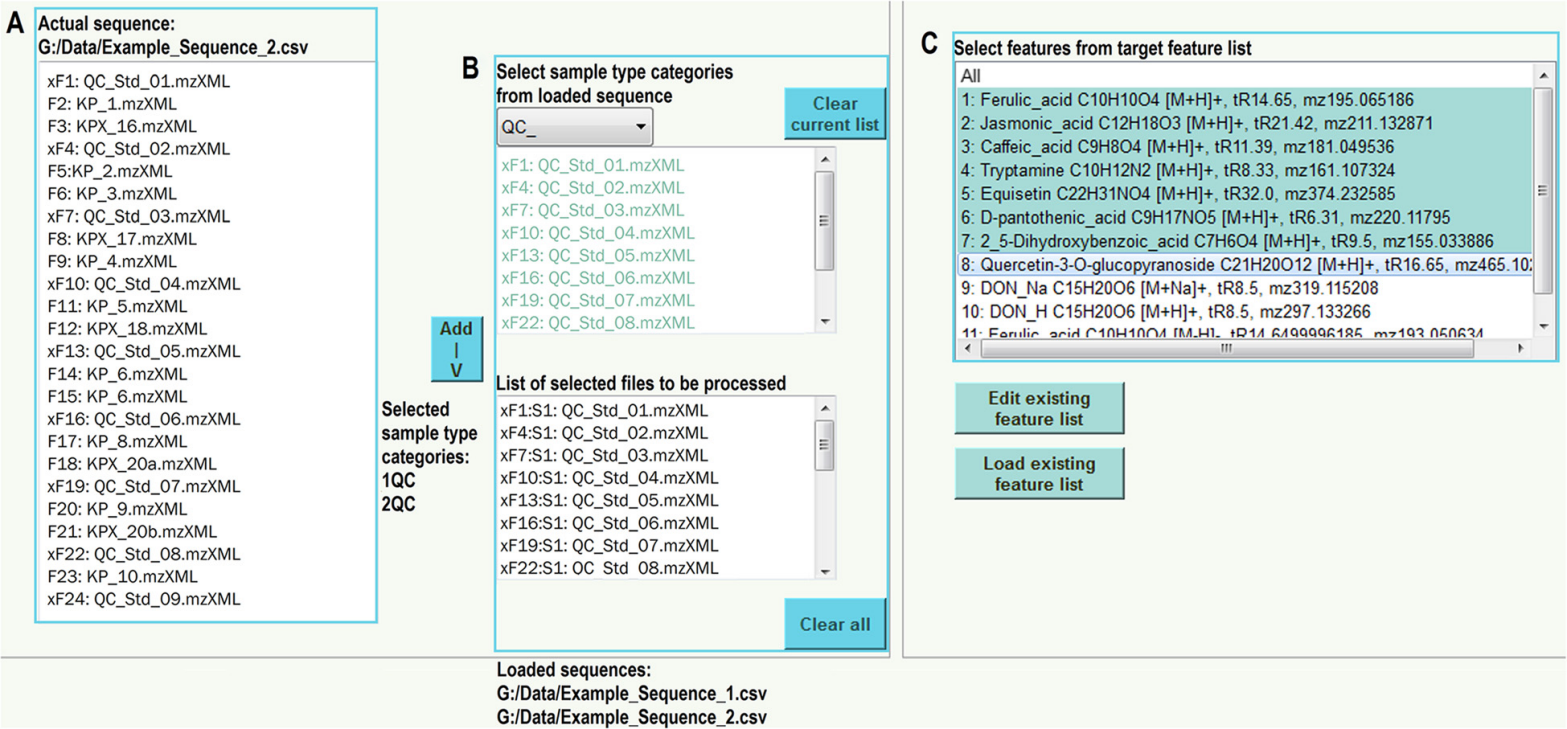
User interface of QCScreen. In **a** the data files contained in the loaded sequence file are listed. In the upper box in **b** the sample type categories can be added to the selected categories in the list below. The target features for evaluation can be defined in **c**

With the software, the user is able to load LC-HRMS data files which must be listed in comma separated value (csv) format, see Fig. 1a). For this, the LC-HRMS raw data first have to be centroided and converted to mzXML format, which can be done with several open-source software tools, e.g. MSConvert [17]. Typically, such a list may consist of a measurement sequence with data files for biological samples and QC samples periodically measured between the biological samples. Based on their file names, QCScreen automatically generates different sample type categories (e.g. blank QC, matrix QC or biological sample) (Fig. 1b). For this, file names must have a unique separator symbol marking the cutoff point to group the files for further data processing (see Additional file 1). The program allows the analysis of multiple sample type categories.

### Target feature definition

Within a local table, target features, of which the quality-related parameters should be checked, are managed. A table view within the GUI allows to add, modify or delete target features. It is also possible to load existing target feature lists, which must be provided in a certain tabular format. Selected target features are then considered for further automated analysis by QCScreen. For every feature, at least an expected tR, the ion species and the target *m/z* value or molecular formula are required, see Fig. 1c.

### Selection of processing parameters and evaluation criteria

For all QC related parameters the software tool offers default parameter settings. Before data processing is started, quality parameter settings can be adjusted. The GUI offers a menu to adjust settings for tR (±sec.) and *m/z* deviation (±ppm) tolerance windows, which are used for chromatographic peak picking via a wavelet implementation [18].

Additionally, for every target feature the parameters tR, *m/z*, EIC feature area and the associated tolerance windows for classification into four quality categories have to be specified. For this, either predefined fixed target values, or alternatively, data-based experimental arithmetic mean, relative bias and standard deviation of the respective feature parameter values can be used, around which the tolerance windows are constructed for performance classification.

### Results generated by the software

When the software finished calculation, a coloured quality overview and for every evaluated performance criterion, a graphical illustration of the generated results is created. For each evaluated parameter i.e. extracted ion chromatograms (EICs), tR, mass accuracy and feature area, the results are plotted against the order of the data files. Moreover, settings e.g. for different scaling of x-axis according to chronological or real (acquisition) time of data files can be adjusted by the user.

For every parameter, an illustration consisting of plots for the parameter values per feature for all processed samples is depicted. For later reference and data evaluation, a list containing further data with the respective arithmetic mean, standard deviations and the calculated parameter values is available in tabular output format. Additionally, the predefined tolerance windows are plotted and for feature area, tR and *m/z* values, box plots are generated to illustrate the precision of these parameters per sample type category.

### Coloured quality overview

The coloured evaluation overview offers an easy-to-interpret illustration of the specified performance criteria (i.e. target value ± tolerance limits, Additional file 1). Feature area precision, tR precision and mass accuracy are shown for evaluated features in the respective data files. Additionally, the results are flagged with four different colours ranging from green (parameter is within expected values) to red (parameter is not within expected values) to provide a visual impression of the overall analytical data quality according to the preselected performance criteria. The purpose of this illustration is to enable an immediate identification of problematic features, parameters or samples. The evaluation overview is arranged as a table of features (rows) and samples (columns). For each entry in this table, the quality-related parameter values are calculated. The retention time parameter values - tR (min), which were experimentally found for the respective features in the evaluated samples, and the tR deviation (min) to the tR specified in the target feature list or to the calculated average tR are displayed. Next to it, the mass parameter *m/z* of the feature found in the sample and the mass accuracy (±ppm) relative to the predefined standard mass or to the calculated average mass are given. At last, the feature area found in the sample and the relative bias for every feature in the respective sample, are displayed. A summary of the average parameter values per feature is given at the right end of the matrix.

### Extracted ion chromatogram (EIC), relative isotopolog abundance (RIA) and feature area illustration

The EIC is defined as the feature intensities at a certain *m/z* (±ppm) value which is plotted as a function of retention time. For each target feature an overlay of all EICs, one per data file, is displayed. This illustration facilitates the visual assessment of the peak profile of the inspected feature(s) across all processed LC-HRMS data files. Optionally, the relative isotopolog abundances (RIA) for Carbon are calculated as the ratio of the ^13^C monoisotopic MS peak to the first isotopic MS peak ^12^C. The graphical illustration showing the accuracy of the experimentally derived RIA and its bias can help to decide whether the data is suitable for sum formula calculation from the experimental RIA values. It should be noted that depending on the mass analyser in use and the mass of the inspected molecule, the resolving power of a mass spectrometer may not allow to completely resolve the isotopic fine structure of the isotopologs under investigation. For sum formulas containing a high proportion of heteroatoms such as N, O or S, the calculated RIA can therefore be biased. To obtain a reliable prediction of the elemental composition this has to be considered. Please also refer to Additional file 1, page 24 for an example.

In the feature area illustration, the integrated area under the chromatographic peak of the respective feature is plotted against the measurement order. This plot can help detect changes or instability of the chromatographic process, MS detector drifts or feature area offsets between data files within or across multiple measurement sequences.

### tR illustration

For every evaluated feature, the determined retention time of the EIC peak is plotted against the different samples to illustrate the chromatographic stability. If specified in the target feature list, different ion species originating from the same metabolite are displayed in parallel within one plot to visually check for their agreement and stability of retention time.

### *m/z* value and mass accuracy illustration

QCScreen generates plots depicting the measured *m/z* (±ppm) values of a chromatographic peak as well as their arithmetic mean and standard deviation against the processed data files. With this type of illustration, the mass accuracy (±ppm) of a specified target feature against the given standard *m/z* or the calculated average *m/z* is determined and represented. These data can for example also be useful for the selection of input parameters for other LC-HRMS data processing software (e.g. XCMS [19]) or a later database search.

## Results

Biological experiments often include measurements over more than one measurement sequence. For time series experiments involving several biological replicates per time point, organism and condition, measurements can extend over several days or even weeks, as was the case for the presented biological experiment, which also served as basis for the development of QCScreen. In such cases, parameters like retention time, mass accuracy and feature area may drift or shift due to changes in MS instrument sensitivity or chromatographic separation. QCScreen has been developed to monitor stability and to promote a comparative analysis of all results over multiple measurement sequences.

### Demonstration of QCScreen with a wheat metabolomics experiment

QCScreen was tested with several data sets measured in short time intervals as well as prolonged time periods over several months. In this example a set of QC standards (Table 1) which had been measured regularly within two different measurement sequences (1 and 2) was processed. The measurement of both sequences was part of a biological experiment which aimed to investigate the metabolic response of two different wheat lines (genotype 1 and genotype 2) after treatment with the mycotoxin deoxynivalenol (DON). For a more detailed description of the biological experiment and LC-HRMS measurements refer to Additional file 1.

**Table 1:** List of authentic reference standards used for preparation of the QC standard

#	Standard	CAS number	Sum formula	Calc. mass	tR (min)
1	Ferulic acid	537–98–4	C_10_H_10_O_4_	194.0579	14.6
2	Jasmonic acid	77026–92–7	C_12_H_18_O_3_	210.1256	21.4
3	Caffeic acid	331–39–5	C_9_H_8_O_4_	180.0423	11.3
4	Tryptamine	61–54–1	C_10_H_12_N_2_	160.1000	8.33
5	Equisetin	57749–43–6	C_22_H_31_NO_4_	373.2253	32.1
6	D-pantothenic acid	137–08–6	C_9_H_17_NO_5_	219.1107	6.31
7	2,5-Dihydroxybenzoic acid	490–79–9	C_7_H_6_O_4_	154.0266	9.47
8	Quercetin-3-O-glucopyranoside	482–35–9	C_21_H_20_O_12_	464.0955	16.6

Each of the tested LC-HRMS measurement sequences contained numerous replicates of blanks (solvent samples), standard QC and pooled biological matrix QC samples which had been interspersed between 75 biological samples. In total, approximately 120 LC-HRMS measurements were carried out per sequence.

### Result plots exemplified with the compound jasmonic acid

**Fig. 2:**
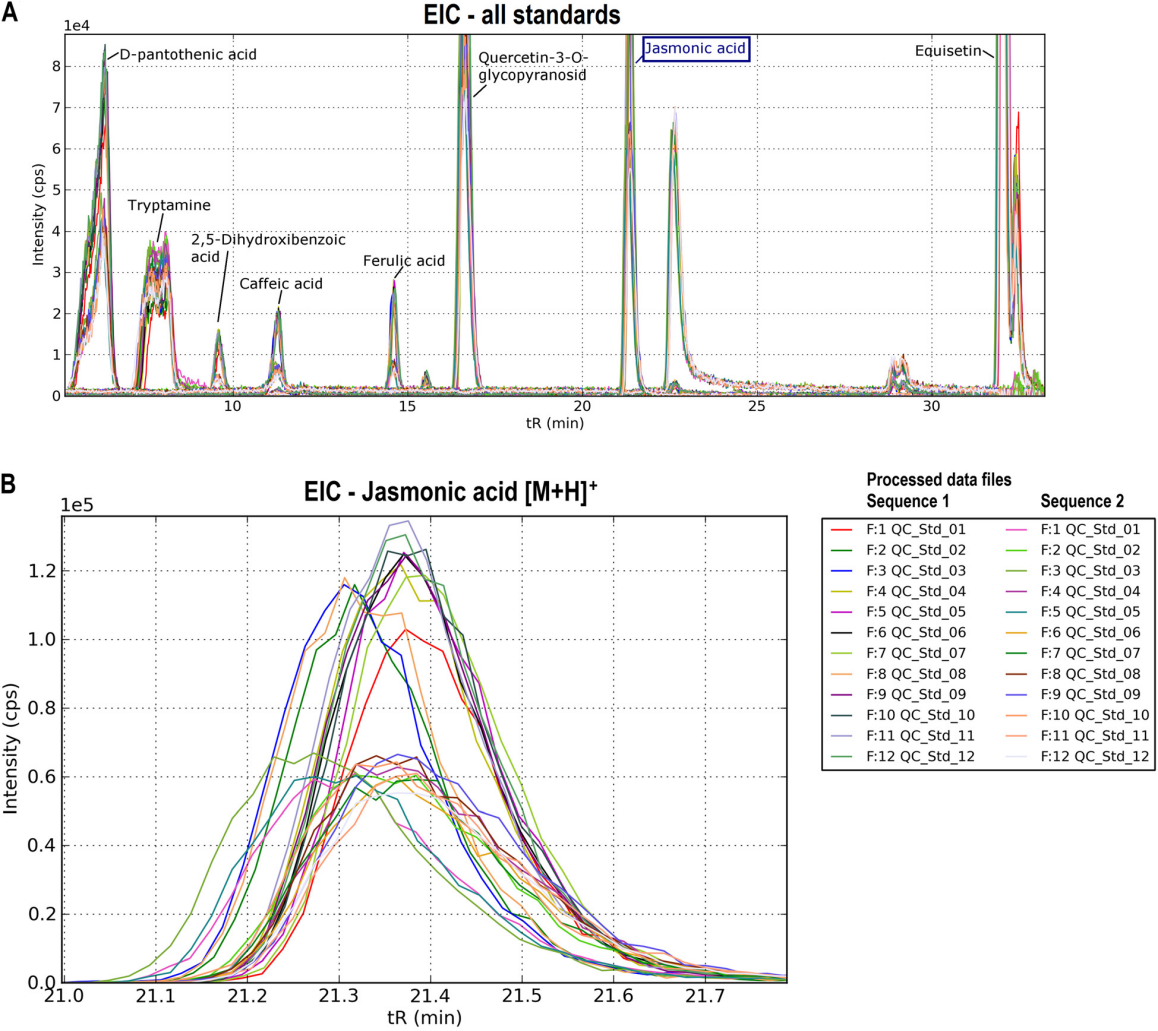
Illustration of EIC overlay of all tested compounds and the selected compound jasmonic acid. The EIC overlay of all tested standard compounds was plotted as retention time in minutes against a function of intensity in counts per seconds, see **a**. The illustration of the particular standard compound jasmonic acid in **b** clearly shows peak separation, chromatographic peak shapes and peak heights. The legend on the right specifies the processed data files in the two measurement sequences whereas F stands for file followed by the number of the file and the name

Evaluation results of QCScreen, generated of a set of standard compounds (Fig. 2a), are described in more detail at the example of a selected standard compound, namely jasmonic acid with the sum formula C_12_H_18_O_3_, eluting at a chromatographic retention time of 21.4 min. Graphical result illustration is given for the ion species [M + H]^+^ with the *m/z* 211.1329 (see Fig. 2b).

For the *m/z* and retention time parameter the predefined values (theoretical *m/z* and expected retention time) were compared to the measured values while for feature area the default setting was to compare the arithmetic mean of all measured feature areas within a particular LC-HRMS sequence with the respective individually measured values, since there was no given theoretical value for feature area available. According to the chosen tolerance limits, each result plot comes with four distinct colour zones from green via yellow and orange to red with the aim to facilitate quality assessment. For assessing mass accuracy of jasmonic acid related LC-HRMS features these tolerance limits had been set as follows: ≤ ±3 ppm (green), ±(3 to ≤ 5) ppm (yellow), ±(5 to ≤ 8) ppm (orange) and > ±8 ppm (red). For more details regarding tolerance windows of retention time, feature area bias and feature area precision refer to Additional file 1, which also provides details with respect to data import and all other parameter settings.

The EIC overlay of all standard compounds in the tested files obviously highlighted the variation of the repeated chromatographic separation and the detector sensitivity across a total of 2 × 12 measurements of the QC standard sample. The EIC overlay for jasmonic acid (Fig. 2b) includes one peak group at 0.6 × 10^5^ and one approximately at 1.2 × 10^5^. This graphical illustration clearly revealed a detector sensitivity shift between the two measurement sequences 1 and 2.

**Fig. 3:**
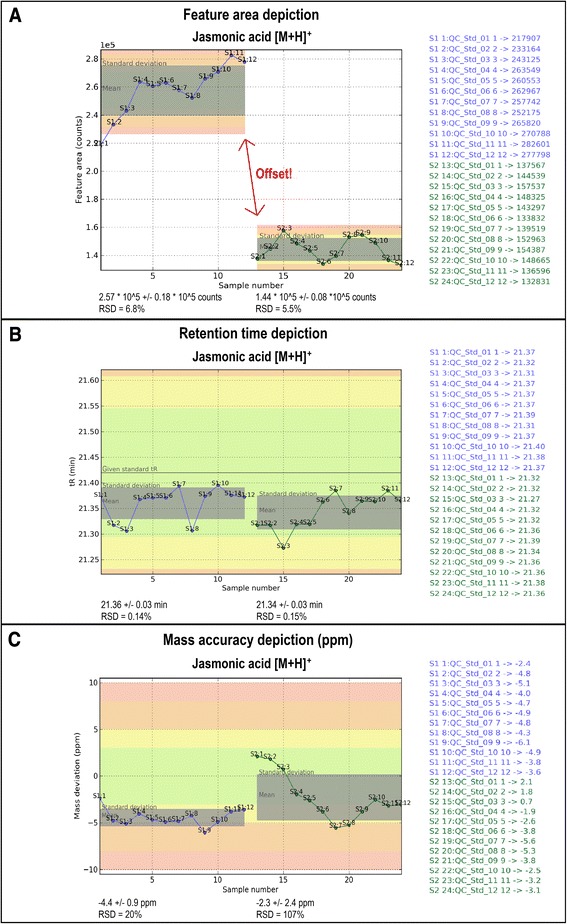
Graphical illustration of feature area, retention time and mass accuracy for jasmonic acid. The illustration of the monitored parameter values of feature area indicates a separation (offset) of the two measurement sequences due to an alteration of instrument sensitivity, **a**. **b** shows a stable chromatographic process and **c** clearly illustrates a stable *m/z* offset in S1 and an *m/z* drift in S2, which can also be seen in the other compounds of the tested standard QC sample (Additional file 1)

This issue can also be investigated more closely in a quantitative manner in the feature area depiction (Fig. 3a). The MS detector sensitivity has obviously changed by a factor of approximately 2 over time in which the two sequences were measured. However, it is also shown that the measured feature areas of the first measurement sequence appeared to be less stable than of the second and steadily increased with the measurement order. Due to the 2-fold sensitivity change observed for the QC standard it is not possible to directly compare absolute feature abundances between the two LC-HRMS sequences. Thus, the comparison between different experimental conditions is not straight forward in such a case. Instead, the data suggest to normalize each of the sequences separately e.g. with the average abundance of the respective features observed in the QC standard or pooled matrix QC sample. Next to sensitivity shifts, deviating data points, like the first and the last value in sequence one can be observed. If a parameter value falls out of the predefined intervals, it also gets flagged with red colour in the coloured overview plot (Additional file 1). The measured retention time allows for a quantitative comparison of samples between the two measurement sequences. In both of the tested sequences, the retention time of jasmonic acid appeared to be slightly lower compared to the user-specified retention time (given tR), Fig. 3b. The graphical illustration of tR indicated a stable chromatographic process over all samples within and between the two measurement sequences.

The illustrations of *m/z* and mass accuracy (±ppm) plotted against the measurement order (see Fig. 3c) showed a higher deviation from the exact mass in the first sequence but overall the mass values within the first sequence showed a higher precision than those of the second sequence. The mass accuracy depiction can be helpful when annotating unknown compounds. Considering just the mass deviation of the first sequence, which ranged between −3 and −5 ppm, based on a −5 ppm tolerance window, compounds measured at the beginning of sequence two might be missed when performing database search. Therefore it is necessary to have a comparative view of the *m/z* and the mass accuracy of all measurement sequences. In sequence two a continuous shift of the mass deviation of jasmonic acid was observed, starting from positive mass deviation decreasing to negative values, which finally increased again. The same mass trends over the two sequences were observed for all tested standards indicating a substance independent behaviour of the MS instrument (see also Additional file 1). In such a case, the values of *m/z* could be corrected with the help of a correction function. The observed mass accuracy in ± ppm may also be used to facilitate the selection of an appropriate mass window for other evaluation software or a later database search.

Evaluation of the RIA showed a stable ratio between the monoisotopic ^12^C MS peak and the first isotopic MS peak ^13^C and indicated that the data are suited for subsequent estimation of the number of carbon atoms per formula unit (see Additional file 1).

### Verification of QCScreen results

To verify that data processing by QCScreen yields consistent and valid results, automatically generated data were compared to manually derived EIC peak area, retention time as well as mass accuracy. To this end, the LC-HRMS raw data of the above described QC standards were also evaluated by the Thermo Xcalibur software. For a detailed comparison between QCScreen and manually derived results see Additional file 1.

## Discussion

Under ideal conditions such as thoroughly selected parameters for sample preparation and LC-HRMS measurements as well as highly stable instrument performance, only minor random fluctuations can be expected to contribute to the overall data variability observed in a metabolomics study. In practice however, the aim to capture as many metabolites as possible in a single measurement is reflected by the use of generic, less accurate technical workflows, which can finally result in significant contributions to both the overall variability as well as bias of the LC-HRMS data. This can also be exemplified with the outcome of the above illustrated study. It is obvious that a combined data evaluation of the biological experiment across the inspected measurement sequences is not straight forward and requires additional processing of data such as normalization of every measurement sequence e.g. by the use of pooled matrix QC samples. For biological interpretation it is important to consider raw data of the complete biological experiment. Otherwise, compounds could possibly be missed, be wrongly annotated or lead to falsely classified as being significantly differing between experimental conditions.

Compared to existing QC evaluation methods embedded in workflows or comprehensive software tools that perform complex algorithms e.g. to reduce analytical variation by automated QC batch correction, QCScreen was developed based on conventional QC charts and enables QC checks without complex statistics. The software tool computes basic performance criteria calculated from the parameter values retention time, feature area and *m/z* value and illustrates the data compared to predefined constraints. For every parameter, the user is able to adjust tolerance limits to define how much the measured values are allowed to deviate from the given values to be still considered acceptable.

As exemplified above, QCScreen allowed an immediate identification of problematic features, parameters and samples. A closer look at the results generated by QCScreen emphasized outliers and offsets and revealed a change of instrument sensitivity as well as mass accuracy between the investigated measurement sequences.

Other comprehensive software (e.g. [13]) also offer a wide range of analysis tools including QC and multivariate analysis and takes a pre-processed quantitation data matrix containing metabolites and samples as input. Pre-processed data matrices are also required by [14], which allows inspecting data for inconsistency or drifts. In contrast, QCScreen does not require prior feature extraction and peak area integration, but directly starts from the LC-HRMS raw data. QCScreen is flexible and allows for comparison of any number of features within one or multiple sequences and multiple sample types within these sequences. The presented software tool is suited to process centroid LC-HRMS full scan data in the mzXML format [16] and automatically extracts and integrates target features. Thus, QCScreen can be used with many different LC-HRMS platforms and aims to assess quality-related parameters of any of this kind of data. For future applications it would be desirable to extend QCScreen by implementing an option for automated correction functions of experimental shifts of retention time and mass accuracy.

## Conclusion

In state-of-the-art metabolomics studies, the development and application of appropriate quality control measures such as QC data processing algorithms are more and more recognized to be crucial prerequisites for obtaining comparable results over longer time periods, across different sample type categories and biological experiments and also between different laboratories [20]. In this respect the presented QCScreen software was developed to process LC-HRMS data sets originating from metabolomics studies and aims to offer a tool for quick visual, easy-to-interpret and descriptive inspection of basic data quality-related parameters such as the stability of chromatographic peak shape, retention time, MS detector sensitivity and mass accuracy. Based on a predefined list of target features, the program can be used to process both multiple QC sample types as well as other experimental samples. Thus, QCScreen provides a good basis for assessing data suitability or to identify the need for developing and applying corrective functions prior to time consuming, detailed data processing and subsequent statistical analysis.

## Availability and requirements

Project name: QCScreen

Project home page: The current version and sample data are available at https://sourceforge.net/p/qcscreen online.

Operating systems: Windows XP, Windows 7

Programming language: Python, R (version 2.12)

Other requirements: The R Project for Statistical Computing, Version R 2.12.0, http://www.r-project.org

License: GNU GPL

Any restrictions to use by non-academics: no restrictions

## Additional file

Additional file 1:
**Contains a description of the biological experiment and LC-HRMS measurements and the exemplification of QCScreen by a step-by-step example including the selection of the data, sample types and target features together with a detailed description of the used parameters, the generated results and a verification of the program.** (PDF 4705 kb)

## Abbreviations

csv: Comma separated value
DON: Deoxynivalenol
EIC: Extracted ion chromatogram
GUI: Graphical user interface
LC-HRMS: Liquid chromatography-high resolution mass spectrometry
*m/z*: mass-to-charge ratio
QC: Quality control
RIA: Relative isotopolog abundance
tR: Retention time
