# Supplementary material for: QCScreen: a software tool for data quality control in LC-HRMS based metabolomics
Source: BMC Bioinformatics. 2015 Oct 24;16:341. doi: 10.1186/s12859-015-0783-x (PMC4619325; doi:10.1186/s12859-015-0783-x)
Supplement: Additional file 1: — Contains a description of the biological experiment and LC-HRMS measurements and the exemplification of QCScreen by a step-by-step example including the selection of the data, sample types and target features together with a detailed description of the used parameters, the generated results and a verification of the program. (PDF 4705 kb) [file 12859_2015_783_MOESM1_ESM.pdf]

Additional file

Content

1. Biological experiment 3
2. LC-HRMS measurements 3
3. Import of data files, QC standards and features 5
4. Parameter settings 9
4.1. General settings 9
4.2. Labelling and style options 9
4.3. Tolerance limits for QC summary 10
5. Results 12
5.1. Colored overview 12
5.2. Graphical illustrations of EIC, feature area and relative isotopolog abundance (RIA) 14
5.2.1. EIC 14
5.2.2. Feature area 18
5.2.3. Relative isotopolog abundance (RIA) 22
5.3. Graphical illustration of tR 25
5.4. Graphical illustrations of mass parameters 29
5.4.1. *m/z* 29
5.4.2. Mass accuracy in +/-ppm 32
6. Validation of the program 35
7. References 39

For every performance parameter QCScreen generates an individual storable tab page which contains the results for the selected features. For later reference an evaluation summary and calculated statistical parameters of all features are combined to a result list and saved in tabular form. The following sections provide a description of the biological experiment and LC-HRMS measurements and the exemplification of QCScreen by a step-by-step example including the selection of the data, sample types and target features together with a detailed description of the used parameters and the generated results.

1. Biological experiment

QCScreen was verified with a set of QC sample types which had been measured at regular intervals within two measurement sequences over several months as part of an untargeted wheat metabolomics experiment. In this experiment a set of QC standards were measured periodically within the two measurement sequences. The aim of the biological experiment was to investigate the metabolic response of wheat lines which exhibit different stages of resistance towards Fusarium head blight (FHB) after the treatment with the mycotoxin deoxynivalenol (DON), comprising the susceptible genotype Remus, the resistant genotype CM and four near-isogenic lines. For more information refer to [1].

Every sequence contained replicates of blanks, standard QC and pooled biological matrix QC between the biological samples which resulted in approximately 120 samples per sequence. QCScreen was tested on the data set and successfully monitored and graphically illustrated parameter performance of selected compounds in the QC standards.

2. LC-HRMS measurements

For evaluation of MS instrument performance with QCScreen, the mixed standard solution was measured at regular intervals by LC-ESI-HRMS. The standard mix, dissolved in methanol/water, (1+1, v+v) contained nine authentic reference standards, which are listed in Table S1 and has been measured periodically after ten experimental samples. Each of the metabolites in the standard mix was measured at a concentration level of 2 mg/L.

#	Standard	CAS number	Sum formula	calc. Mass	Rt (min)
1	**Ferulic acid**	537-98-4	$C_{10}H_{10}O_4$	194.0579	14.57
2	**Jasmonic acid**	77026-92-7	$C_{12}H_{18}O_3$	210.1256	21.42
3	**Caffeic acid**	331-39-5	$C_9H_8O_4$	180.0423	11.3
4	Tryptamine	61-54-1	$C_{10}H_{12}N_2$	160.1000	8.33
5	Equisetin	57749-43-6	$C_{22}H_{31}NO_4$	373.2253	32.05
6	D-pantothenic acid	137-08-6	$C_9H_{17}NO_5$	219.1107	6.31
7	2,5-Dihydroxybenzoic acid	490-79-9	$C_7H_6O_4$	154.0266	9.47
8	Quercetin-3-O-glucopyranoside	482-35-9	$C_{21}H_{20}O_{12}$	464.0955	16.58

Table S1. List of authentic reference standards used for preparation of the QC standard

LC-HRMS measurements were performed as recently described by [2], using an UHPLC system (Accela, Thermo Fisher Scientific, San Jose, CA, USA) coupled to a LTQ

Orbitrap XL (Thermo Fisher Scientific). Ionisation was performed with an electrospray ionisation interface in positive polarity mode. A reversed-phase XBridge C18, 150x2.1 mm i.d., 3.5 µm particle size (Waters, Milford, MA, USA) analytical column, preceded by a C18 4x3 mm i.d. security cartridge (Phenomenex, Torrance, CA, USA) were employed using Methanol and water, both containing 0.1 % formic acid as solvents. The flow rate was held at 250 µL min-1 using a linear gradient program. The chromatographic method held the initial mobile phase composition (10 % B) constant for 2 min, followed by a linear gradient to 100 % B within 30 min. This final condition was held for 5 min, followed by 8 min of column re-equilibration at 10 % B. The MS instrument interface was operated in positive ion mode with the following settings: sheath gas: 60 arbitrary units, auxillary gas: 15 arbitrary units, sweep gas: 5 arbitrary units, capillary voltage: 4 kV, capillary temperature: 300°C. The orbitrap mass analyser was operated in full scan mode in a scan range from *m/z* 100 - 1000 with a resolving power setting of 60,000 FWHM (at *m/z* 400).

3. Import of data files, QC standards and features

First a sequence file is loaded. All files for this example are available at

https://sourceforge.net/projects/qcscreen

located in the folders "example_dataset_1.zip" and "example_dataset_2.zip". Before continuing, all data files that have to be checked must be extracted and located to the same folder than the corresponding sequence file is located. The first sequence file is in the folder "example_dataset_1.zip ". There the sequence table „Genotype_S1_FullSequence.csv“ was opened by clicking on „Load sequence table“.

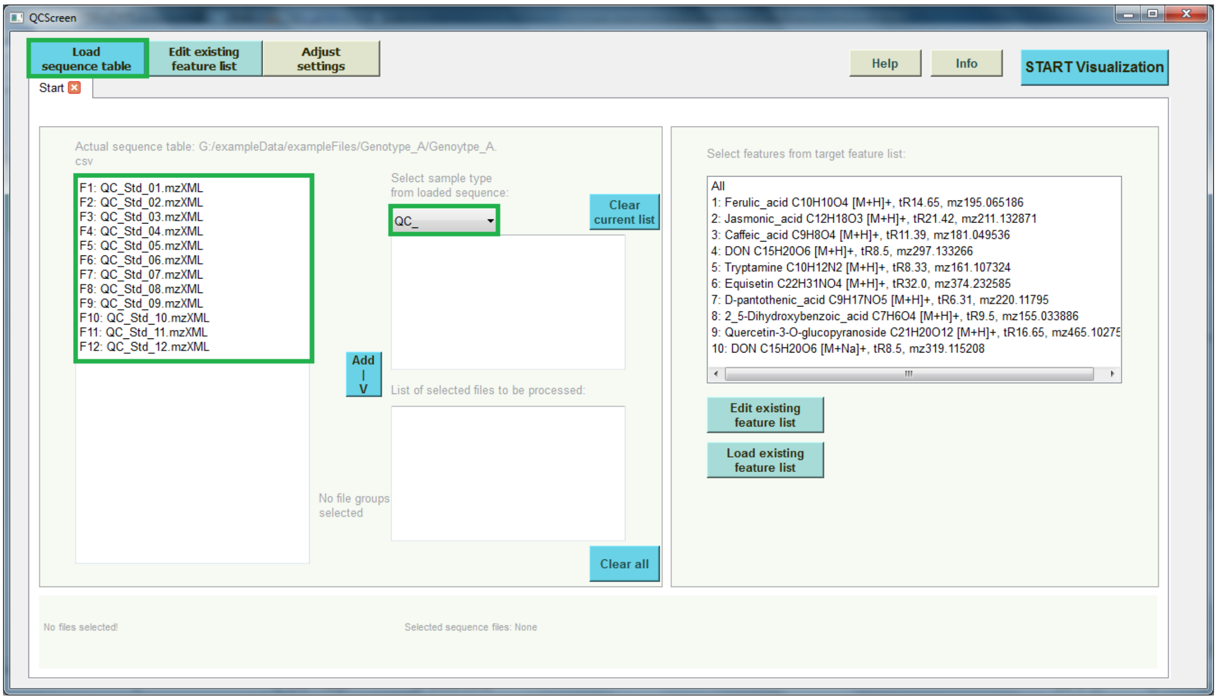

Figure S1. „Load sequence table“

The file „Genotype_S1_FullSequence.csv“ was simply created as a csv file containing the column „File Name“ with the file names of the samples that should be checked.

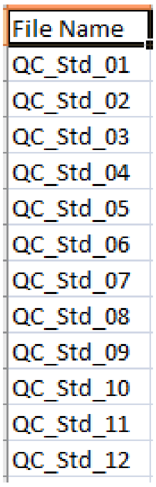

File Name
QC_Std_01
QC_Std_02
QC_Std_03
QC_Std_04
QC_Std_05
QC_Std_06
QC_Std_07
QC_Std_08
QC_Std_09
QC_Std_10
QC_Std_11
QC_Std_12

Table S2. Genotype_S1_FullSequence.csv

It is also possible to load a sequence file containing different sample type categories like blanks, biological samples, pooled biological matrix QC and QC standards but for reasons of simplicity only a list of QC standards was used in this exemplification.

QCScreen then loads the sequence table and generates groups based on the file names. Then file names are separated into groups by means of the separator symbol "_". Now groups can be selected by clicking on the drop down "Select sample type from loaded sequence". In this example only one group "QC_ " is added to the total selection on the bottom with the "Add" button on the left.

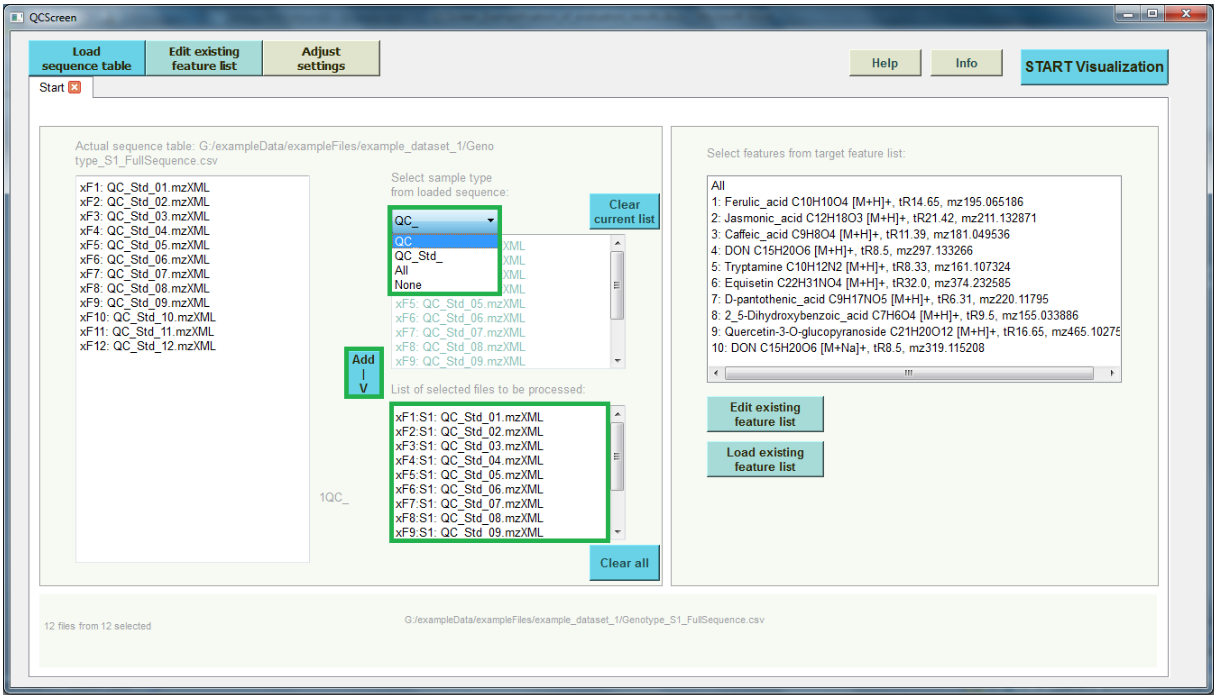

Figure S2. „Select sample type categories“

Another sequence table, "Genotype_S2_FullSequence.csv" in the folder "example_dataset_2“, is then loaded. There the second "QC_" group is selected and added to the actual selection. The next step is to select target features. In this example, target features are selected from the already existing feature list. By clicking on "Load existing feature list" another feature list can be loaded. Other examples for feature lists can be found in the folder „example_featurelist.zip“.

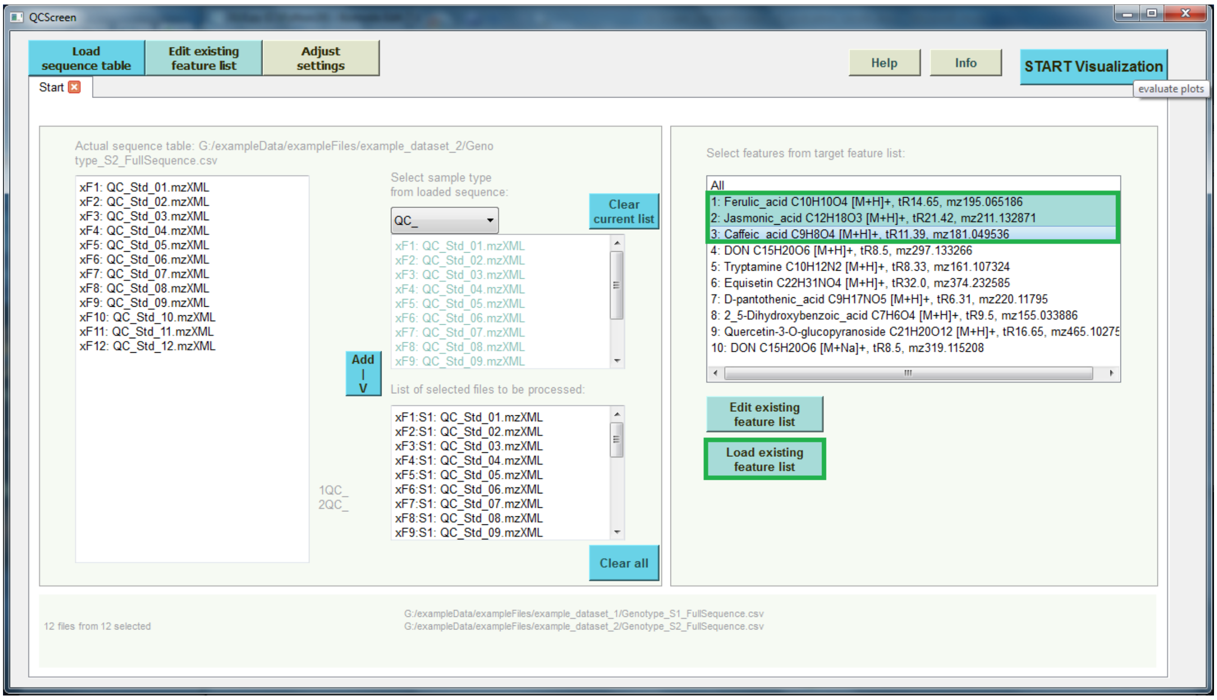

Figure S3. „Select target features"

By clicking on the features, the first three listed features (ferulic acid, jasmonic acid and caffeic acid) are selected as target features (green upper box in Figure S3). The performance parameters associated to these features will then be checked in the selected data files.

Optional, settings can be made. For this example data set, the default settings were adopted, except the setting "Choose results plots to be evaluated". Here the check box for the graphical illustrations of the RIA was checked.

After the configuration of the settings, the QC analysis can be started by a click on the button "START Visualization". The progress bar gives an indication of the actual data processing progress. The overview and the result illustrations are displayed in individual tab pages. The result can be stored as images. Additionally a report file is generated.

4. Parameter settings

For the selected example QCScreen was tested with the following (default) settings.

4.1. General settings

tR tolerance for peak picking

+/-tR (sec.): **30.**

Mass tolerance for peak picking

+/-ppm: **10.**

4.2. Labelling and style options

Mass range

1. Mass range box plots **Not checked** (default)

Choose x-axis style

Equidistant chronological measurement distance of samples **Checked** (default)

Choose result plots to be evaluated

Feature plots (EIC, feature intensity, tR (min), mass accuracy (ppm), *m/z*) are generated **Checked** (default)

RIA plots (ratio M+1/M of monoisotopic 12C and first 13C isotopolog) are generated **Checked** (not default)

4.3. Tolerance limits for QC summary

Default intervals for performance categories of retention time deviation (+/−sec) and mass deviation (+/−ppm) are suggested automatically due to the default peak picking parameter values.

Tolerated retention time deviation (+/- sec)

Target value: **Take given value from feature list** (default)

Color-Intervals	From	To
Green	0	7
Yellow	8	11
Orange	11	15
Red	15	22

Tolerated mass deviation (+/- ppm)

Target value: **Take theoretical value** (default)

Color-Intervals	From	To
Green	0	3
Yellow	3	5
Orange	5	8
Red	8	10

Tolerated feature area bias (%)

Target value: **Take arithmetic mean of measured values** (default, currently only option)

Color-Intervals	From	To
Green	0	5
Yellow	5	7
Orange	7	10
Red	10	12

Tolerated RSD of feature area (%)

Target value: **Take arithmetic mean of measured values** (default, currently only option)

Color-Intervals	To
Green	3
Yellow	5
Orange	7
Red	10

Furthermore it is worth to mention that LC parameters related to sample injection like injection volume, injection solvent composition and others are of great importance considering signal intensity and retention time reproducibility. In order to minimize random deviation in measurement data, it is therefore recommended to select these experimental parameters carefully.

5. Results

All the results shown in the presented experiment were generated applying the above explained parameter settings. QCScreen was applied to standards in the standards list in Table S1. This example demonstrates the application of QCScreen on only the first three plant metabolites, marked blue in Table S1, ferulic acid, jasmonic acid and caffeic acid. All the results can be saved as images. The generated results summary is saved in csv-format.

5.1. Colored overview

The first generated result tab page offers a quick colored overview of data quality based on the user pre-defined performance categories. In the colored matrix it is illustrated at a glance if, based on the chosen criteria, the data files contain potentially problematic features/samples.

In the "results overview" window features are organised in lines, whereas individual samples are displayed columnwise. For every feature-sample pair the calculated parameter values are given in "colored boxes" (colored according to the predefined quality categories). Additionally, for every line and column useful parameters are summed up per sample type category and every measurement sequence per feature respectively. Here, the evaluated parameter values of sequence A are displayed. The last 4 parameter values summarize the results for the evaluated sample type category (in this case over the whole sequence) and the RSD of the feature area per inspected feature and sample type category. Further to the right the next category is displayed (here only parts of the results are displayed).

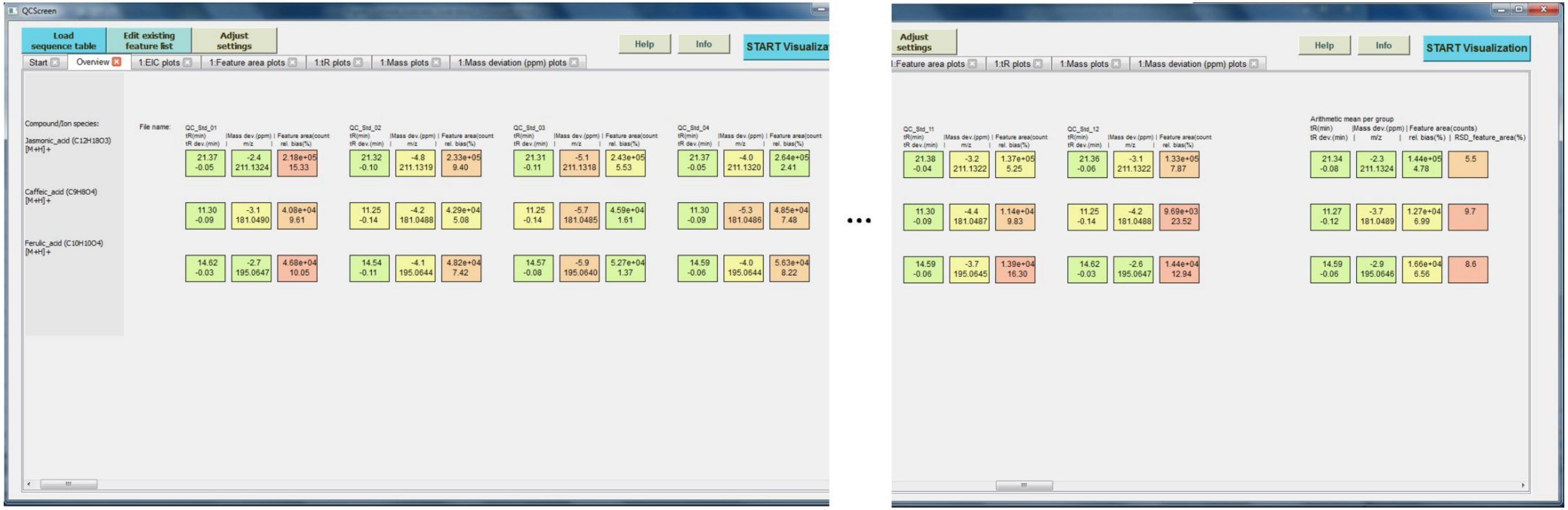

Figure S4. Colored overview window displays tR (+/-sec) and tR deviation from that specified in feature list (+/-min), *m/z*, mass accuracy (+/-ppm), feature area and relative bias values for every compound/ion species – sample pair, RSD of feature areas over groups.

5.2. Graphical illustrations of EIC, feature area and relative isotopolog abundance (RIA)

5.2.1. EIC

The extracted ion chromatogram (EIC) can be defined as intensity of a signal at a certain *m/z* value which is plotted as a function of tR. The EIC chromatograms are overlaid to illustrate the variation of the chromatographic peaks over different samples for single features.

Two different types of plots are generated: 1) An overlay of all EIC chromatograms of the selected compounds, caffeic acid and ferulic acid. 2) An overlay of the EIC of every sample is plotted. The legend shows the file/sample numbers and the file/sample names.

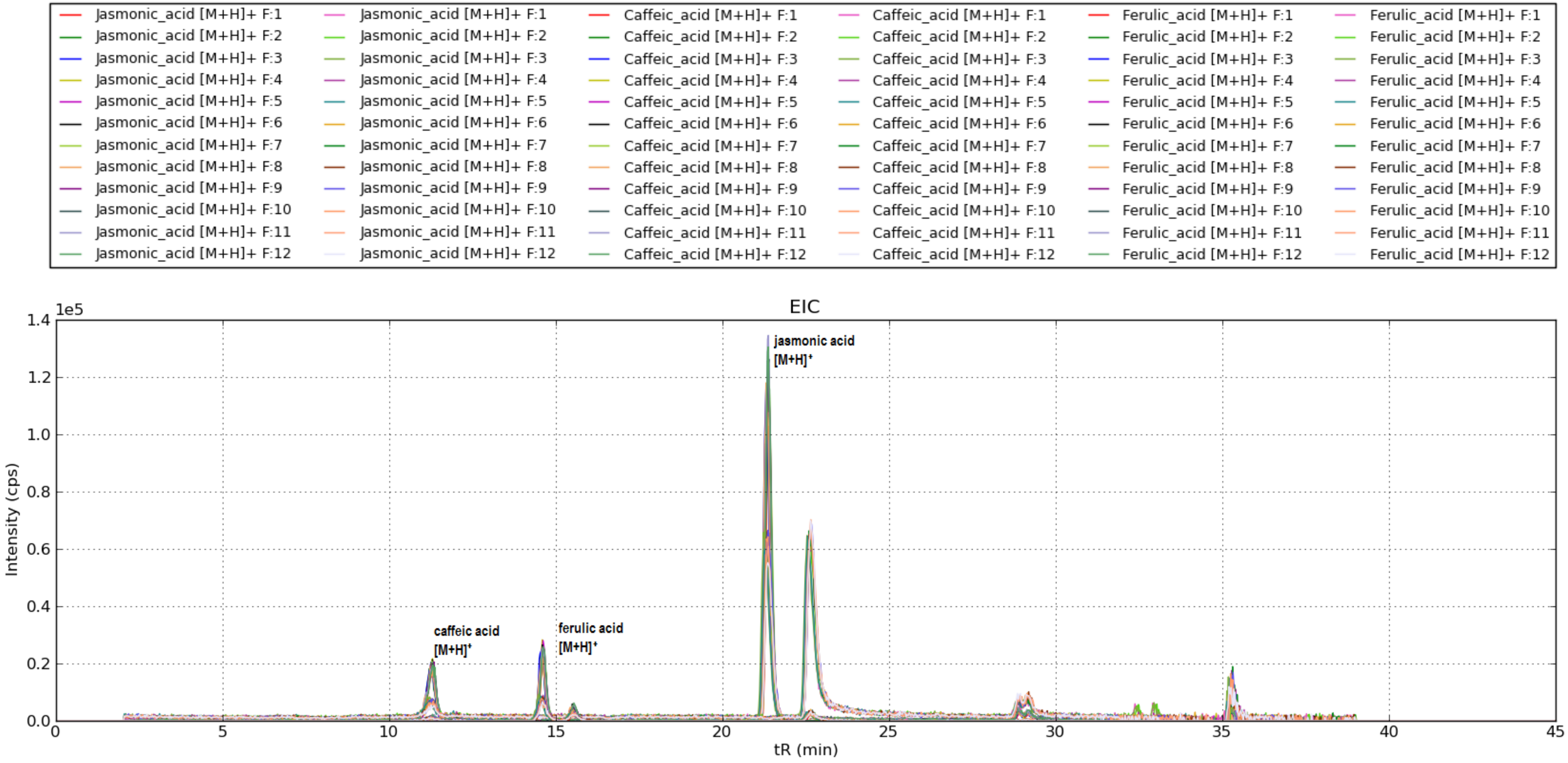

Figure S5. EIC of all selected features (caffeic acid, ferulic acid and jasmonic acid) in the selected measurement sequences (listed in legend above).

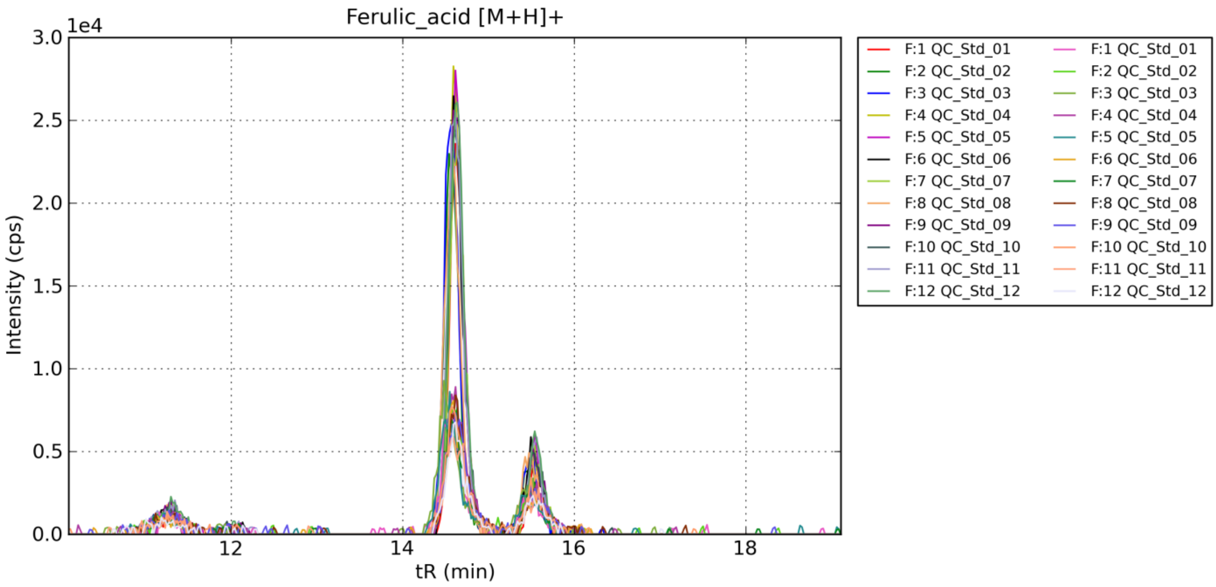

Figure S6. EIC ferulic acid $[M+H]^+$ over all measurement sequences.

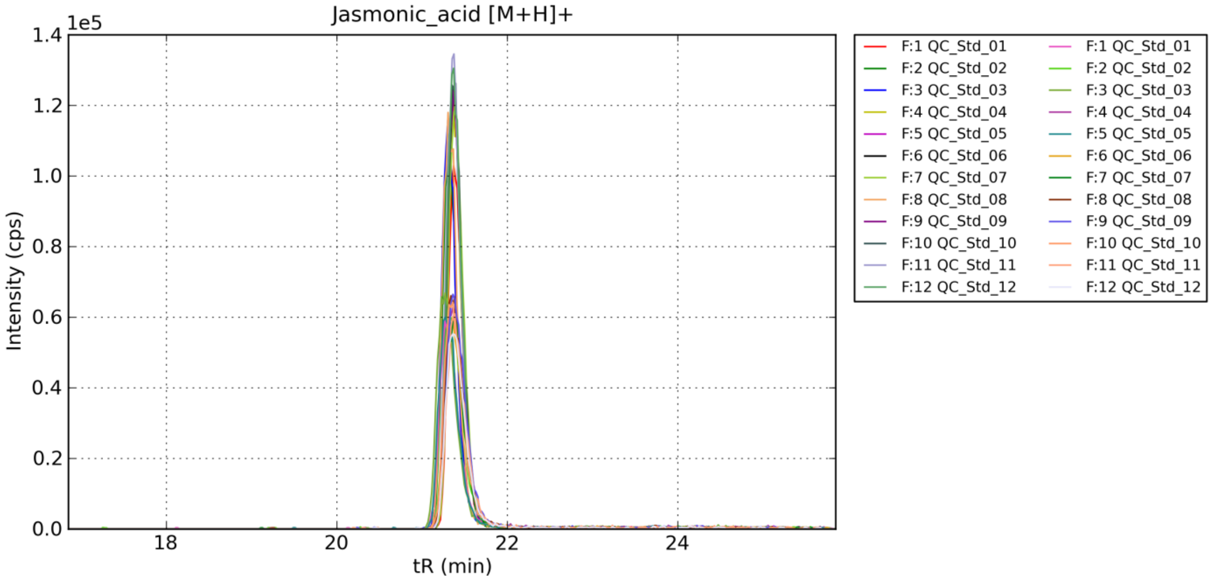

Figure S7. EIC jasmonic acid $[M+H]^+$ over all measurement sequences.

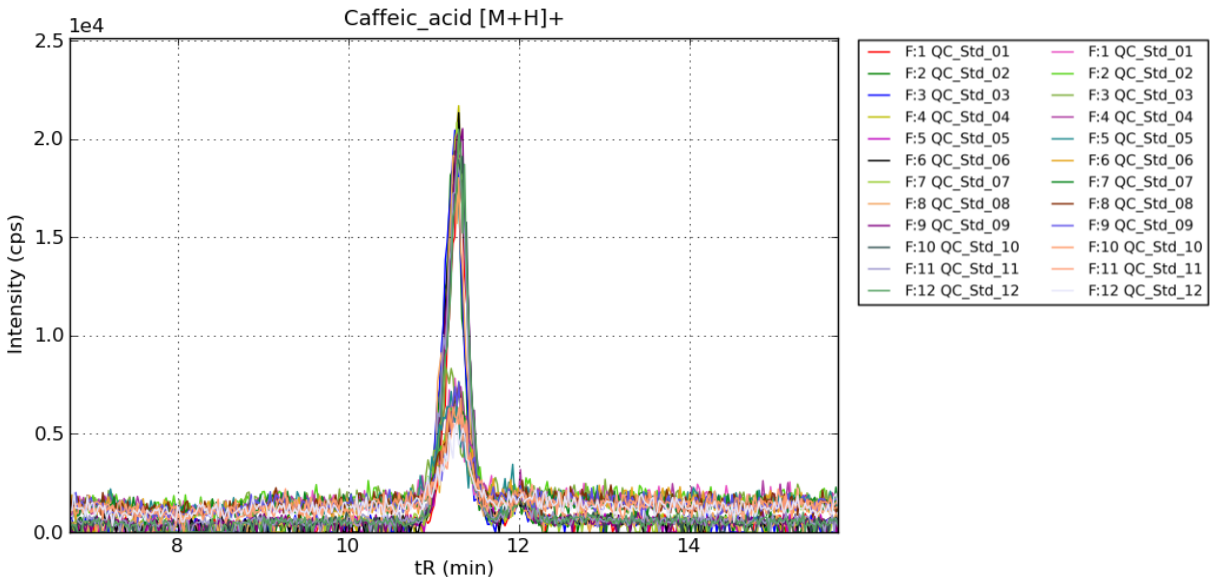

Figure S8. EIC caffeic acid $[M+H]^+$ over all measurement sequences.

5.2.2. Feature area

The illustration for feature area plots in which the integrated area under the EIC peak is plotted against the order of measured samples. The two dashed shaded areas behind each of the data file groups display +/- 1x the standard deviation. Additionally the arithmetic mean for every data file group is plotted within the standard deviation area. The different tolerance limits are displayed as colored areas. Additionally in the second type of illustration, box plots for every feature and data file groups are displayed.

For every feature specified in the target feature list, a separate plot is generated which shows the respective feature areas over all processed LC-HRMS data files. In the illustrated example, results for $[M+H]^+$ of caffeic, jasmonic and ferulic acid are shown from two different measurement sequences. The illustrations show, that for these three compounds the feature areas are higher in the first sequence than in the second sequence. These illustrations can visualize significant instrument sensitivity offsets between the two measurement sequences.

ç

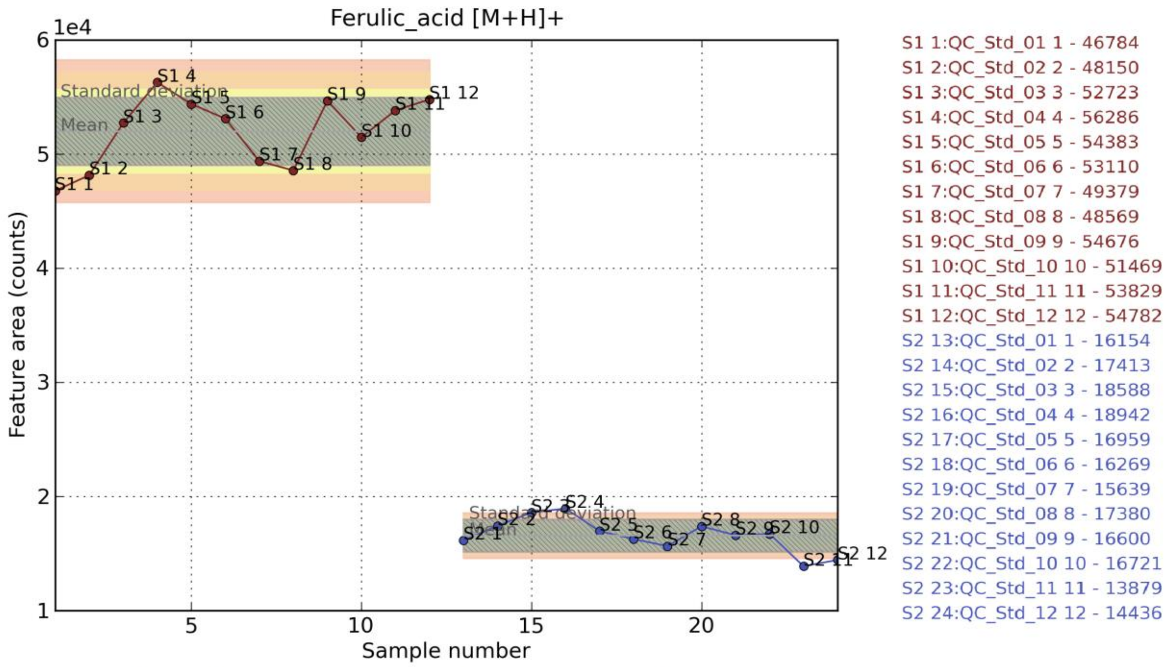

5.20 * 10^4 +/- 0.30 * 10^4 counts
RSD = 5.7%

1.66 * 10^4 +/- 0.14 *10^4 counts
RSD = 8.6%

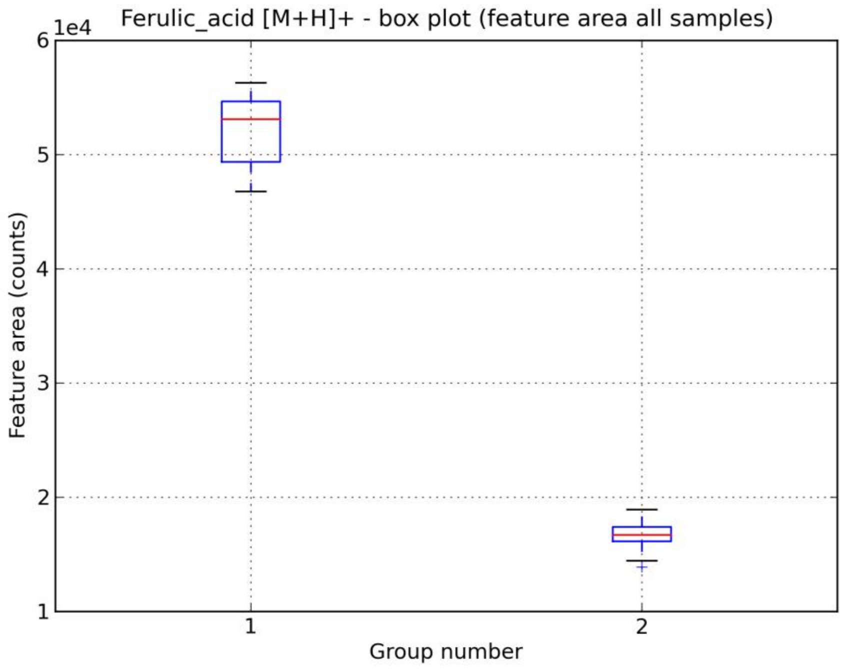

Figure S9. Feature area plots of ferulic acid $[M+H]^+$ plots the first sequence S1 in red with a higher integrated area under the EIC and the second sequence S2 in blue which shows a lower area under the EIC. Corresponding box plots below visualize the scatter of the respective feature areas per measurement sequence.

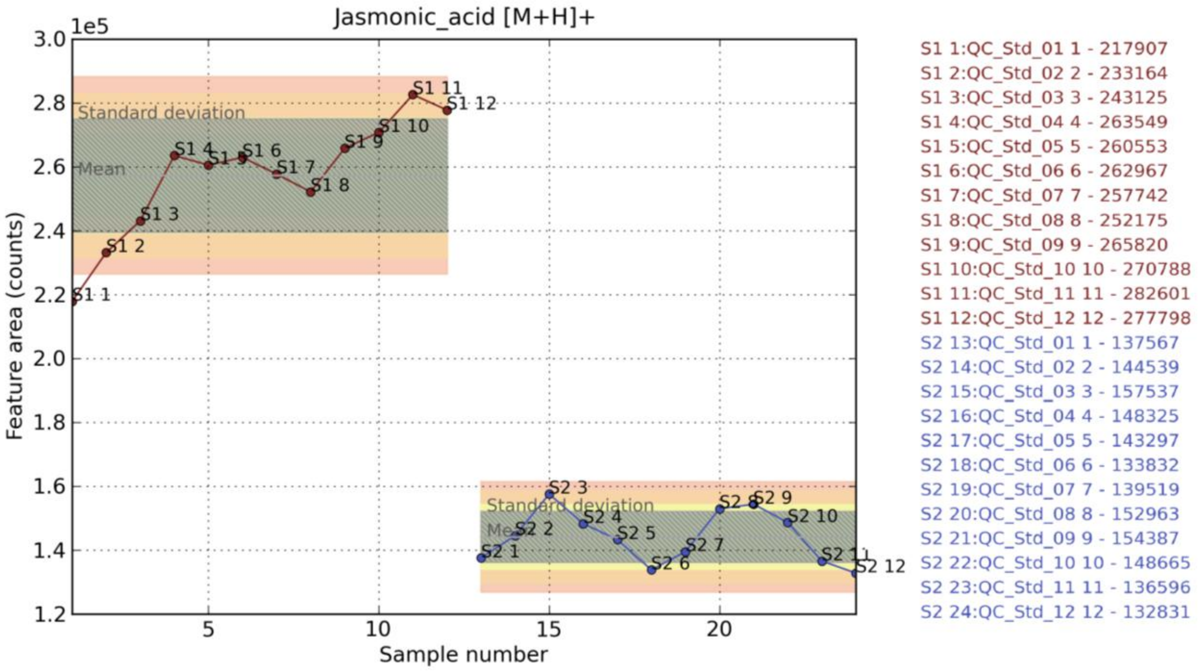

2.57 * 10^5 +/- 0.18 * 10^5 counts
RSD = 6.8%

1.44 * 10^5 +/- 0.08 *10^5 counts
RSD = 5.5%

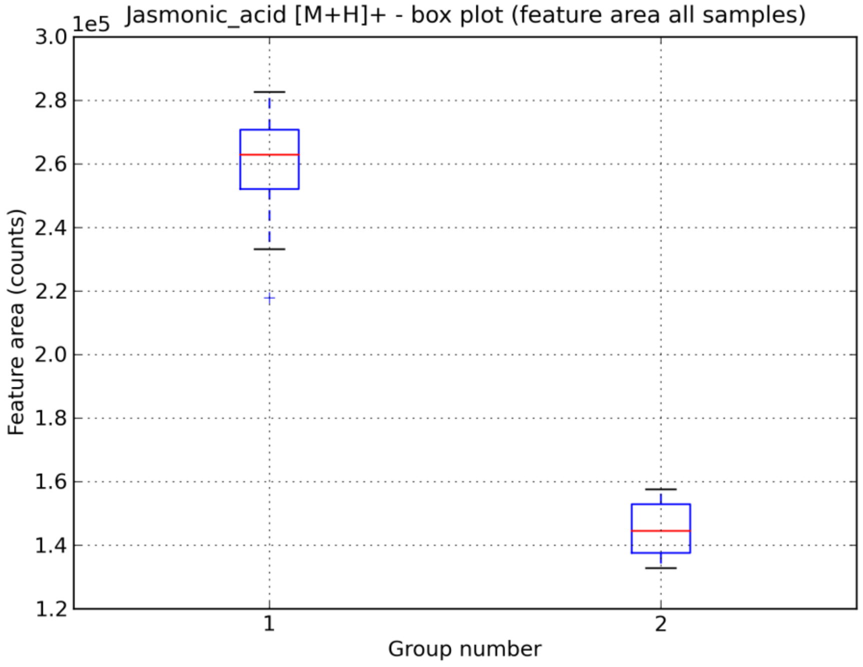

Figure S10. Feature area plots of jasmonic acid $[M+H]^+$ plots the first sequence S1 in red with a higher integrated area under the EIC and the second sequence S2 in blue which shows a lower area under the EIC. Corresponding box plots below visualize the scatter of the respective feature areas per measurement sequence.

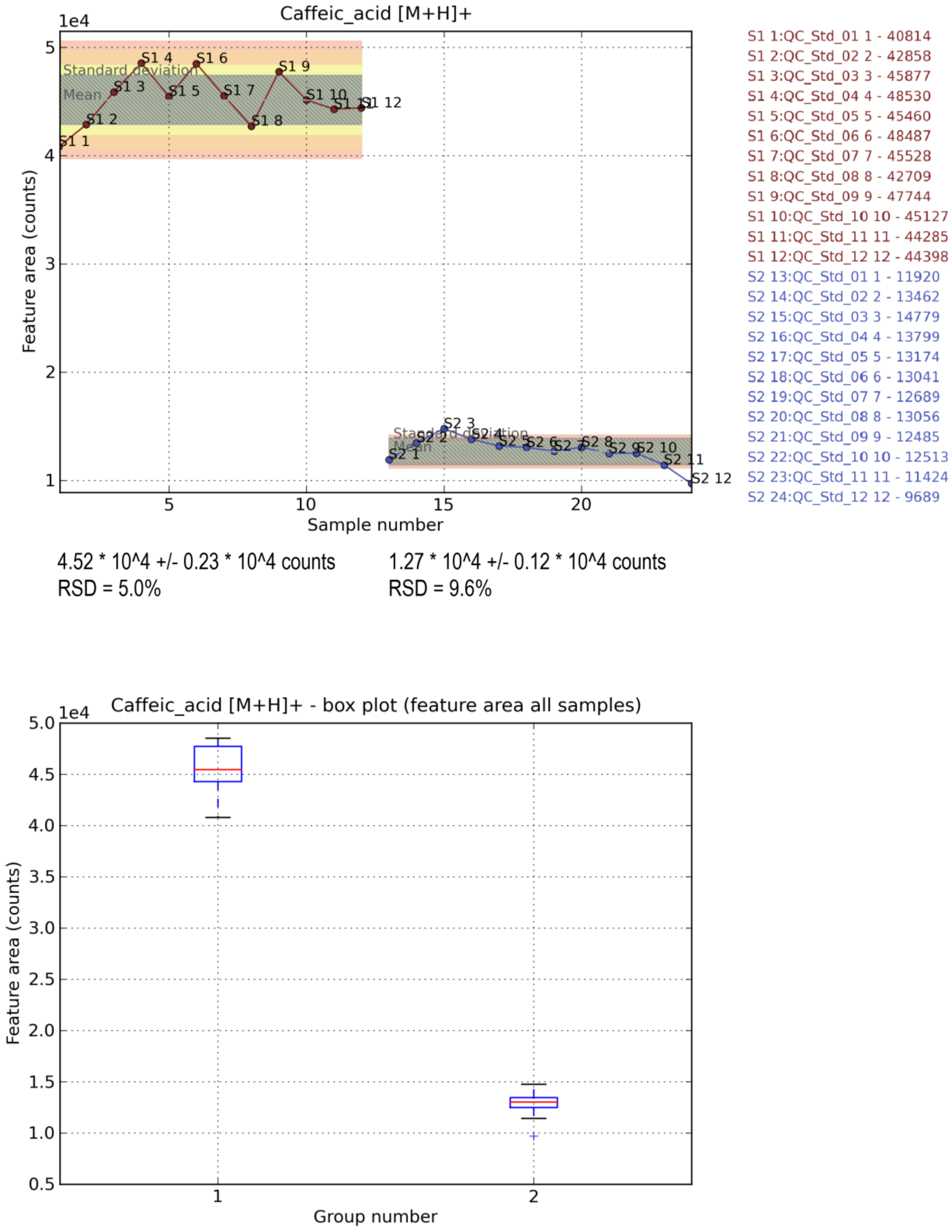

Figure S11. Feature area plots of caffeic acid [M+H]$^+$ shows the first sequence S1 in red with a higher integrated area under the EIC and the second sequence S2 in blue which shows a lower area under the EIC. Corresponding box plots below visualize the scatter of the respective feature areas per measurement sequence.

5.2.3. Relative isotopolog abundance (RIA)

Since the relative RIA can be used to derive e.g. the number of carbon atoms in the ion species under investigation, and to further elucidate the molecular formula of unknown metabolites, the accuracy of experimental RIA values is of interest in many metabolomics applications. QCScreen offers an option to generate RIA illustrations.

The RIA is calculated for every MS scan as the ratio of the respective M+1/M MS peak. If the experimental and the theoretical RIA are not in a certain ratio, it can be indicated that the RIA is not suited for sum formula calculation.

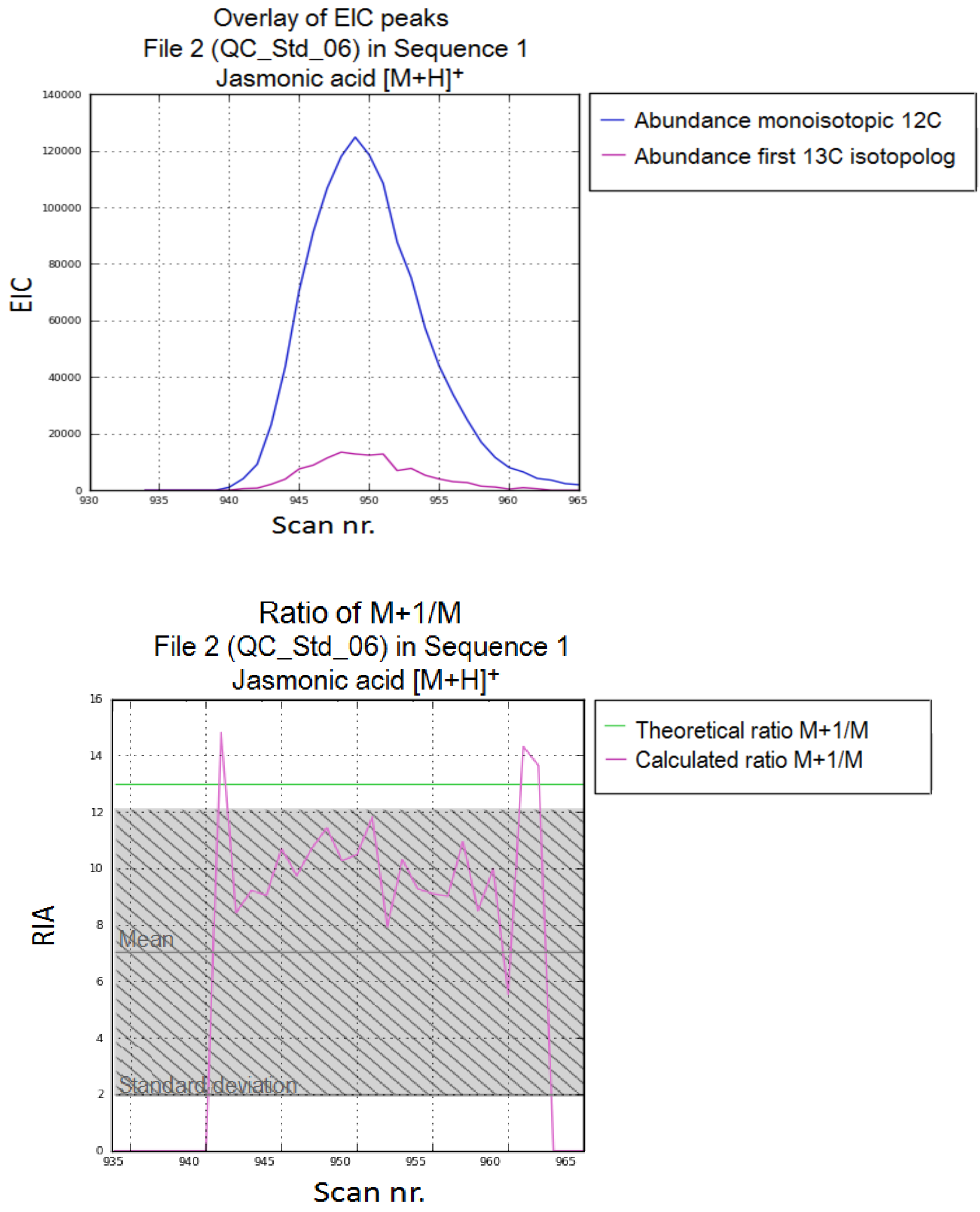

Figure S12. EIC of M and M+1 peaks and relative isotopolog abundance ratio (RIA) for jasmonic acid in file QC_Std_06.

For every MS scan the ratio of the signal height of M+1/M (the monoisotopic ^{12}C and the first ^{13}C isotopolog) is calculated and plotted against scan numbers. The theoretical RIA is depicted in the plot to allow for estimation of the RIA accuracy observed for the inspected feature and sample. In this example, one can see that the calculated (i.e. experimental) ratio of the two MS signal abundancies M+1 and M, is almost always below the theoretical ratio of 16.2%.

The accuracy of the RIA value depends on the resolving power of the mass spectrometer used, which has to be critically considered in practice. Resolving power may range from a few thousand for older TOF instruments via 10 to the power of 5 for Orbitraps to 10 to the power of 6 for FT-ICR MS instruments. For sum formulas with high N or S content the limited mass resolving power may result in only partly resolved isotopic fine structures which can finally lead to biased RIA values in the centroid spectra as shown below for simulated spectra of the protonated molecule for a hypothetical compound with the formula $C_6H_{12}NO_4PS_2$. Here, the first isotopolog M+1 is affected by incomplete resolution of its isotopic fine structure.

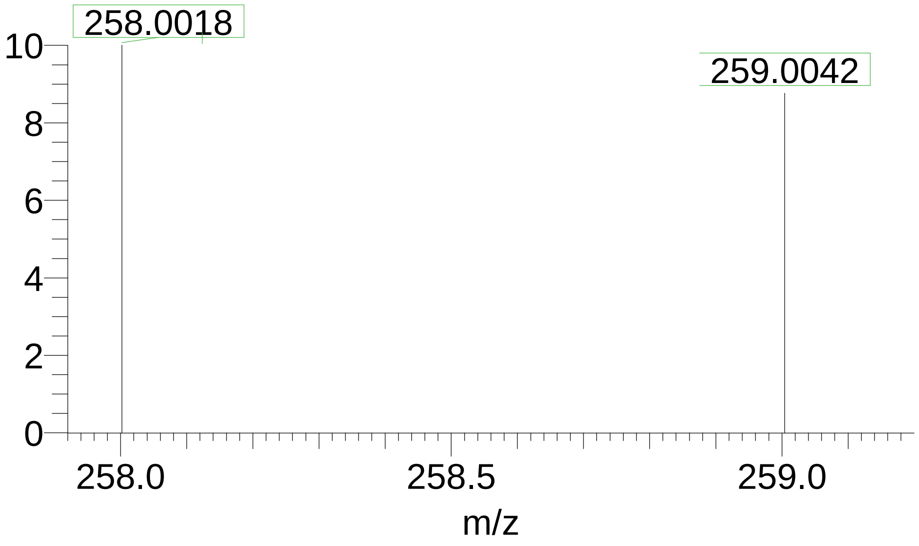

Figure S13. Isotopic fine structure of the protonated molecule with the sum formula $C_6H_{12}NO_4PS_2$ at TOF resolving power of 6000.

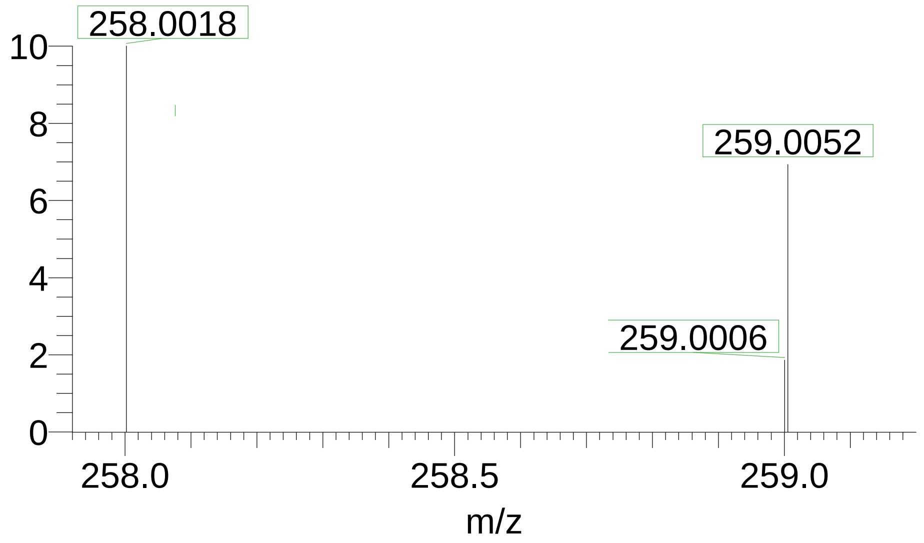

Figure S14. Isotopic fine structure of the protonated molecule with the sum formula $C_6H_{12}NO_4PS_2$ at resolving power of 100000.

5.3. tR

The tR is plotted over the different samples to illustrate the precision of chromatographic retention time across the inspected LC-HRMS raw data files, for the assessment of the stability of the chromatographic process. If specified in the feature list, different ion species originating from a single metabolite will be displayed in parallel within one plot.

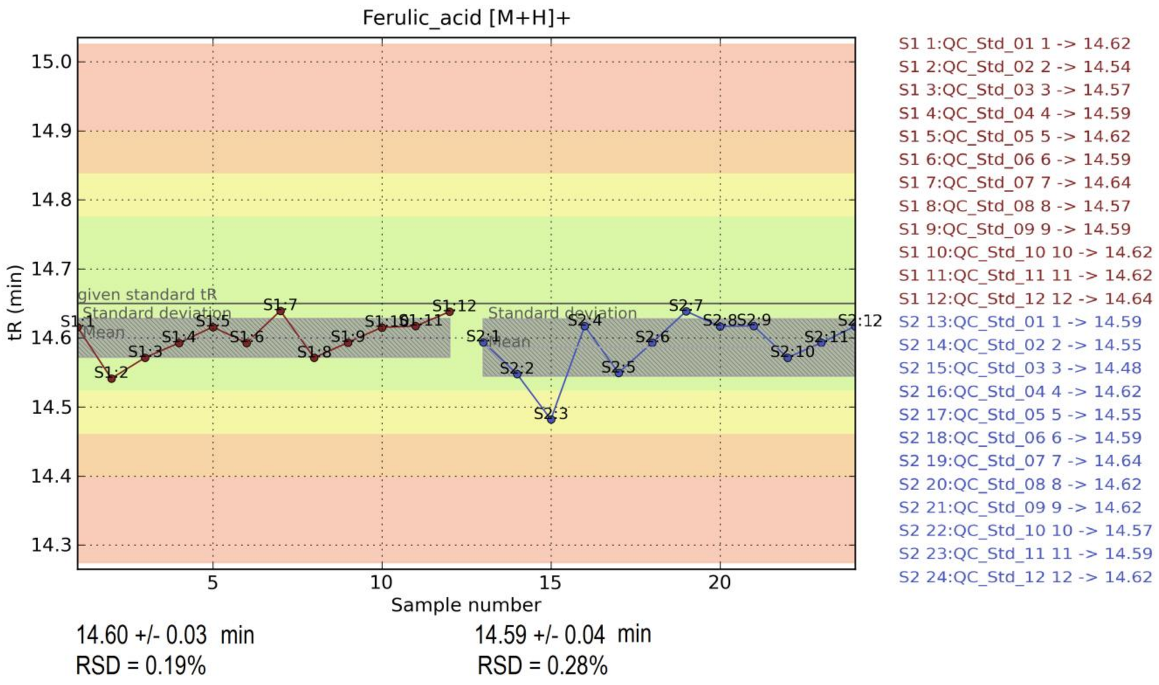

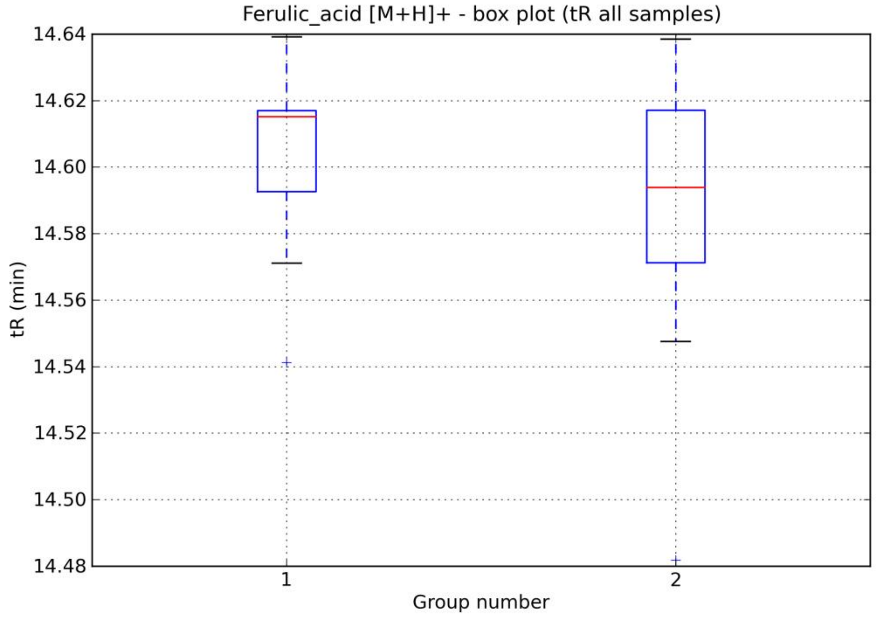

Figure S15. tR plot of ferulic acid [M+H]$^+$ plots the tR (min) of the first sequence S1 in red and the second sequence S2 in blue. Corresponding box plots below visualize the scatter of tR within the two measurement sequences (sequence 1 left with more precise tR than sequence two on the right).

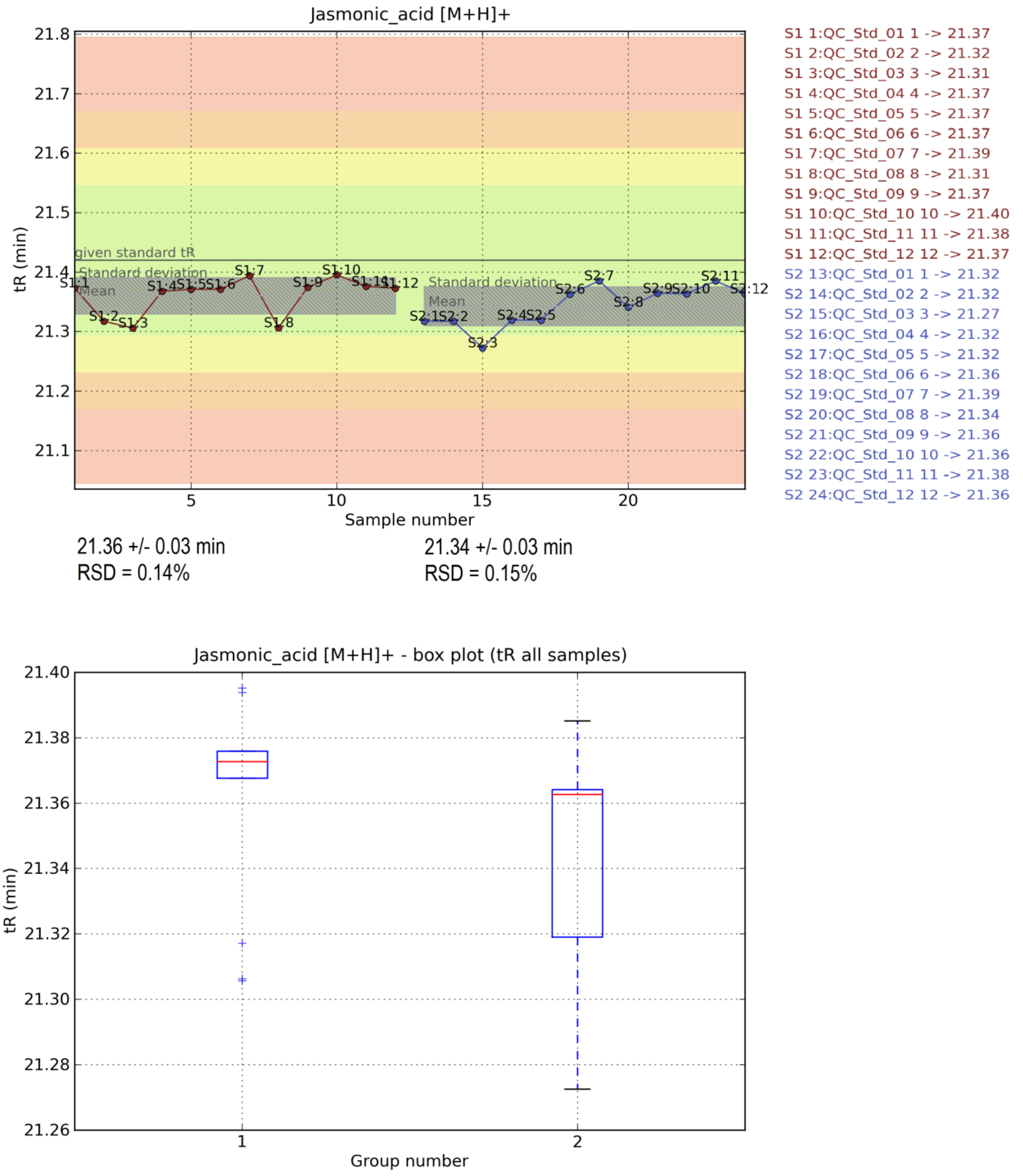

Figure S16. tR plot of jasmonic acid $[M+H]^+$ plots the tR (min) of the first sequence S1 in red and the second sequence S2 in blue. Corresponding box plots below visualize the scatter of tR values within each of the inspected measurement sequences.

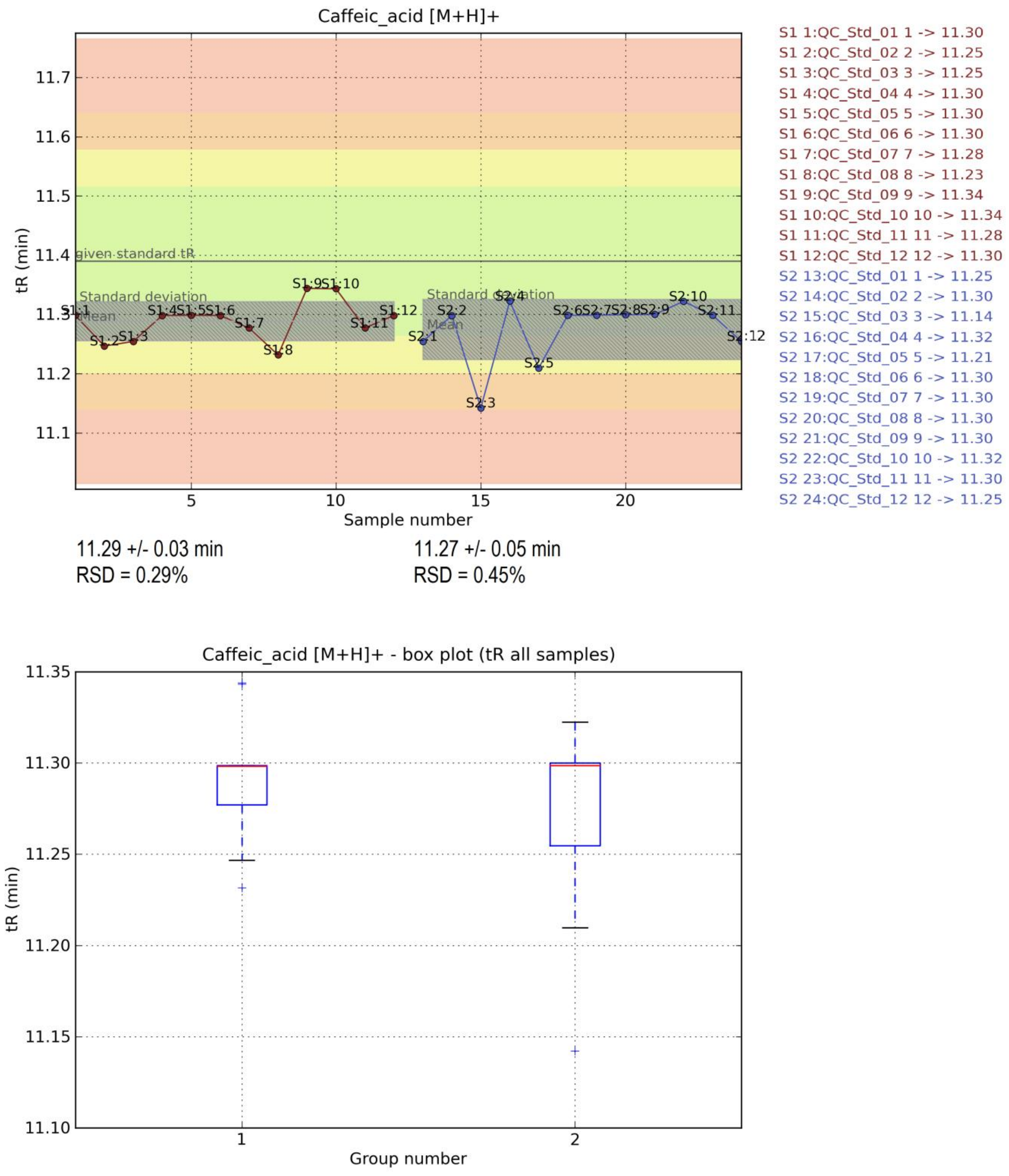

Figure S17. tR plot of caffeic acid $[M+H]^+$ plots the tR (min) of the first sequence S1 in red and the second sequence S2 in blue. Corresponding box plots below visualize the scatter of tR values within each of the inspected measurement sequences.

5.4. Graphical illustrations of mass parameters

5.4.1. *m/z*

This type of illustration shows the *m/z* value plotted against the selected samples in one plot per feature.

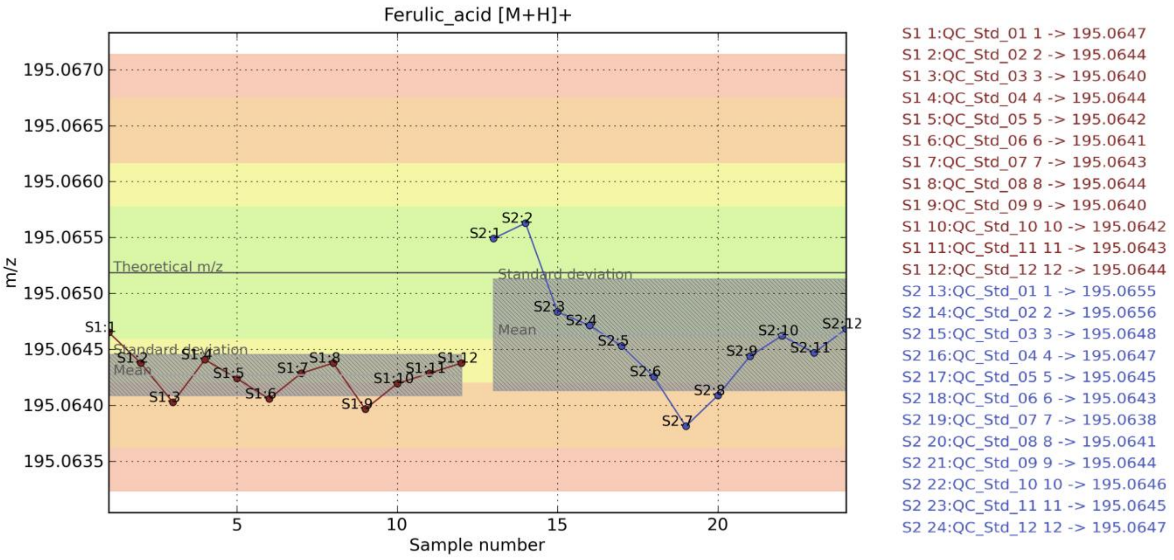

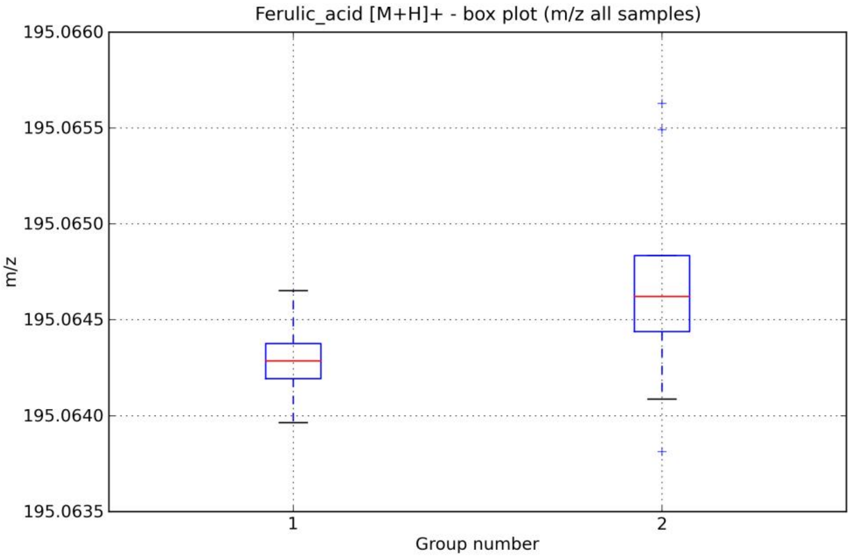

Figure S18. *m/z* plot of ferulic acid $[M+H]^+$ plots the *m/z* of the first sequence S1 in red and the second sequence S2 in blue and corresponding box plots.

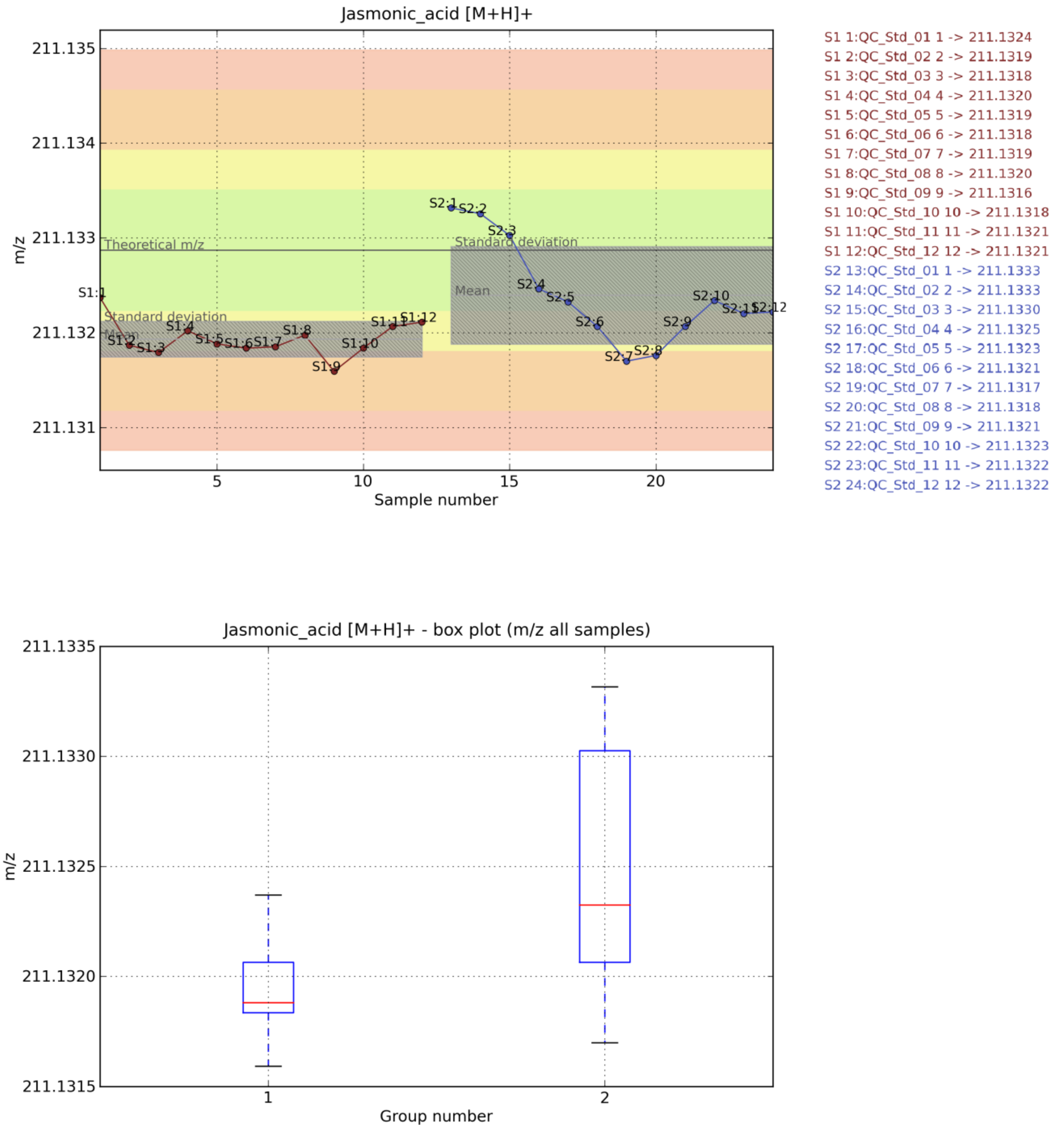

Figure S19. *m/z* plot of jasmonic acid $[M+H]^+$ plots the *m/z* of the first sequence S1 in red and the second sequence S2 in blue and corresponding box plots.

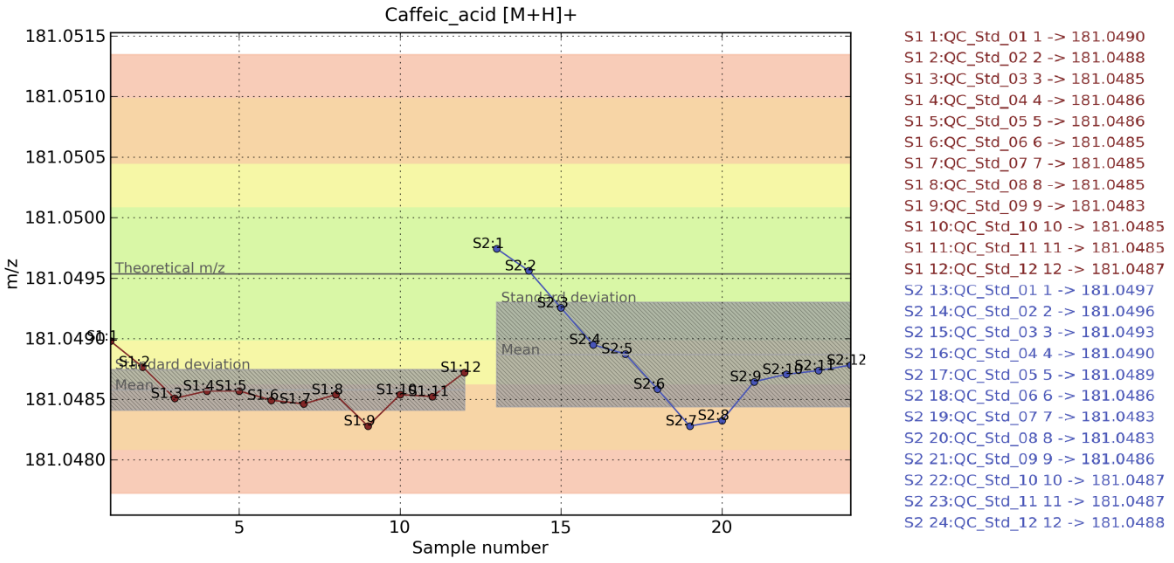

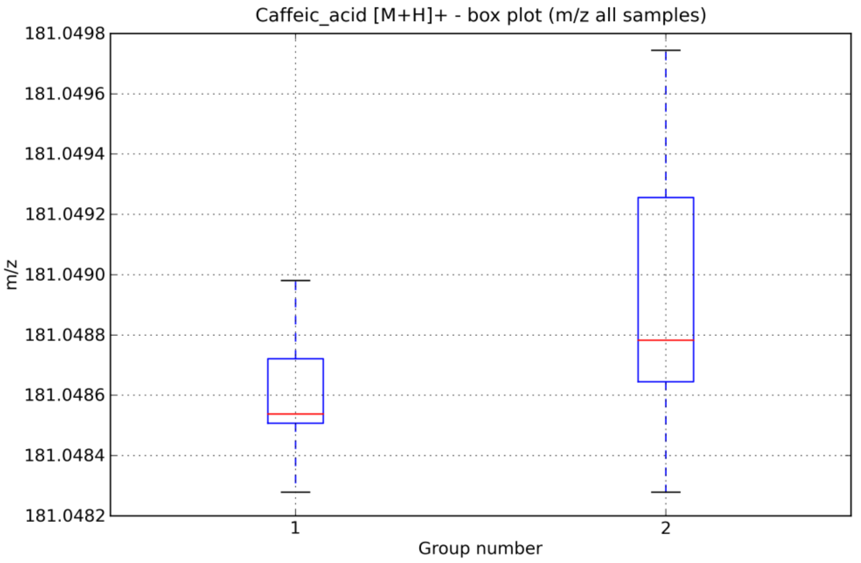

Figure S20. *m/z* plot of caffeic acid $[M+H]^+$ plots the *m/z* of the first sequence S1 in red and the second sequence S2 in blue and corresponding box plots.

5.4.2. Mass accuracy in +/-ppm

In addition to the *m/z* plots the mass accuracy is illustrated as relative deviation of the exact feature mass (+/-ppm). If more than one measurement sequence is evaluated, mean and corresponding standard deviation of the achieved mass accuracy is depicted separately.

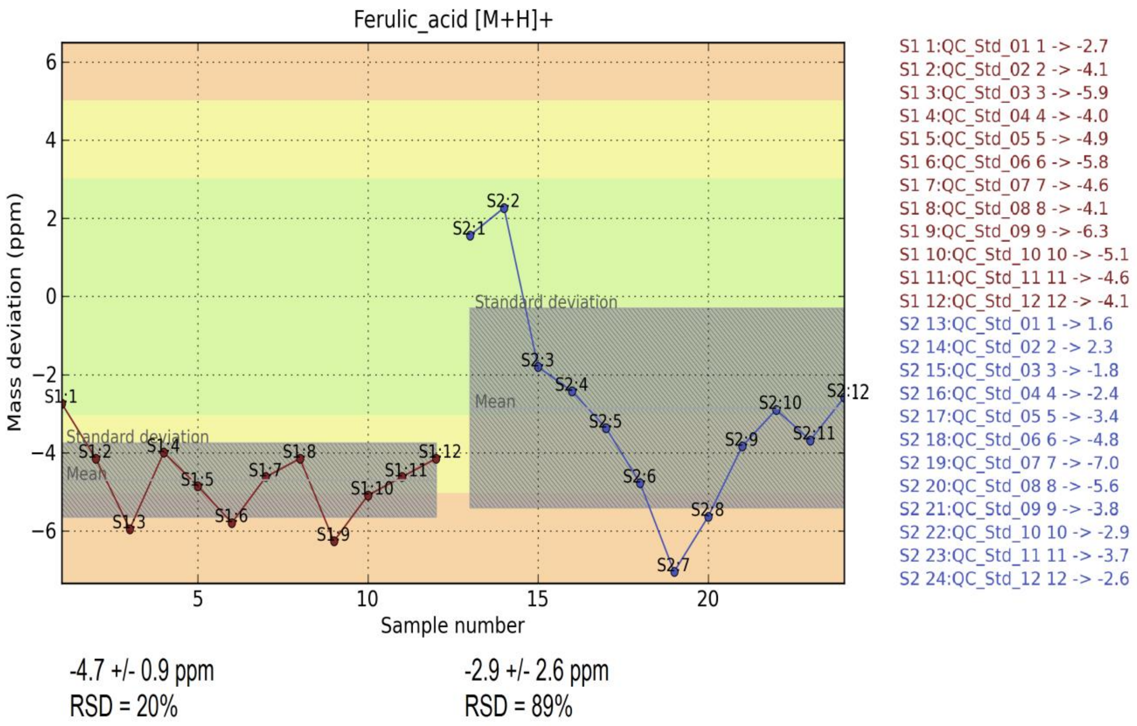

Figure S21. Mass accuracy (+/-ppm) plot of ferulic acid $[M+H]^+$. The relative deviation between measured and exact feature mass (rel. bias) is illustrated for the first sequence S1 (red) and the second sequence S2 (blue).

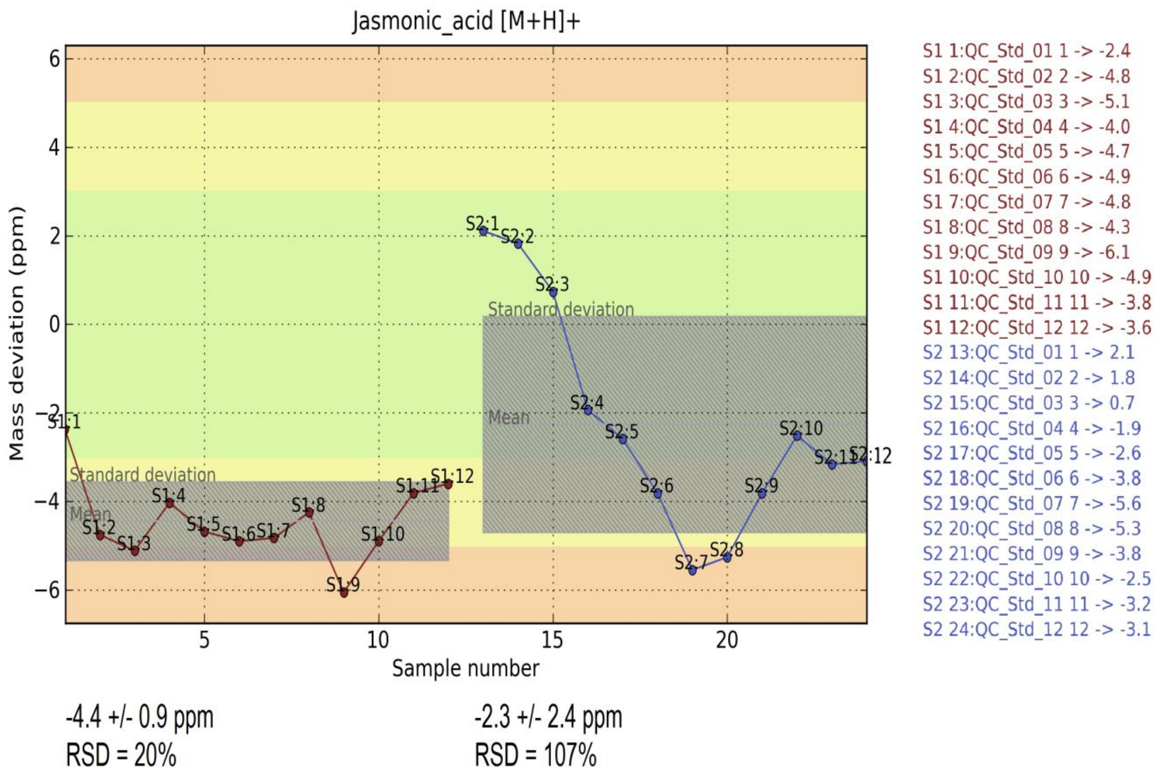

Figure S22. Mass accuracy (+/-ppm) plot of jasmonic acid $[M+H]^+$. The relative deviation between measured and exact feature mass (rel. bias) is illustrated for the first sequence S1 (red) and the second sequence S2 (blue).

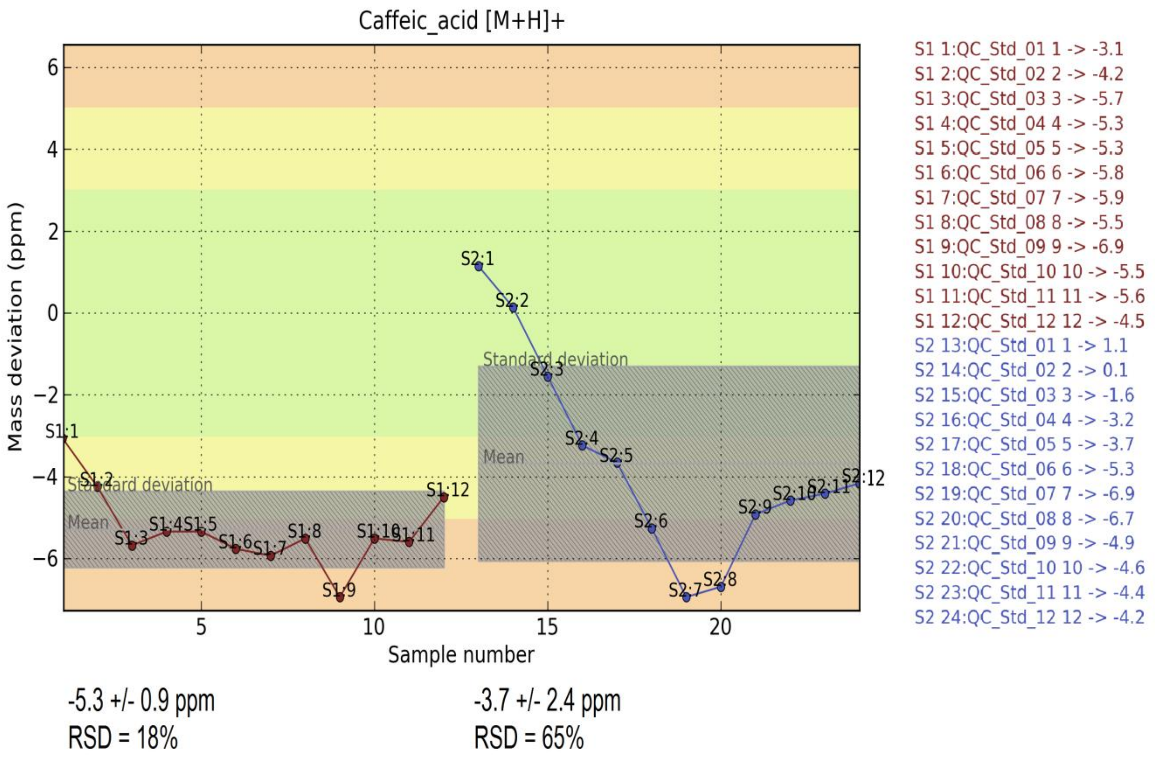

Figure S23. Mass accuracy (+/-ppm) plot of caffeic acid $[M+H]^+$. The relative deviation between measured and exact feature mass (rel. bias) is illustrated for the first sequence S1 (red) and the second sequence S2 (blue).

6. Validation of the program

For verification of results obtained from QCScreen, data evaluation was performed in parallel using Thermo Xcalibur software 2.2 SP1. 48. For manual data processing, ICIS peak detection and integration algorithm was employed for all data files. Processed data were vrified using Quanbrowser and all integrated peaks were inspected manually and re-integrated if necessary. Peak areas and retention times were exported to MS Excel and further processed. Mass accuracy was determined manually in the Qualbrowser by using the most intense scan in the respective extracted ion chromatogram of each standard.

In the following, the manual evaluation of the three parameters feature area, retention time and mass accuracy is opposed to the results generated by QCScreen.

Genotype	Area	Compound<br>Ferulic acid	Jasmonic acid	Caffeic acid
CM	**RSD (%) manual validation**	**7.4**	7.9	5.9
	**RSD (%) QCScreen**	**5.7**	6.8	5
Remus	**RSD (%) manual validation**	**9.3**	6.0	10.6
	**RSD (%) QCScreen**	**8.6**	5.5	9.6

Table S3. RSD for feature area of ferulic acid, jasmonic acid and caffeic acid of manual validation compared to the results generated by QCScreen.

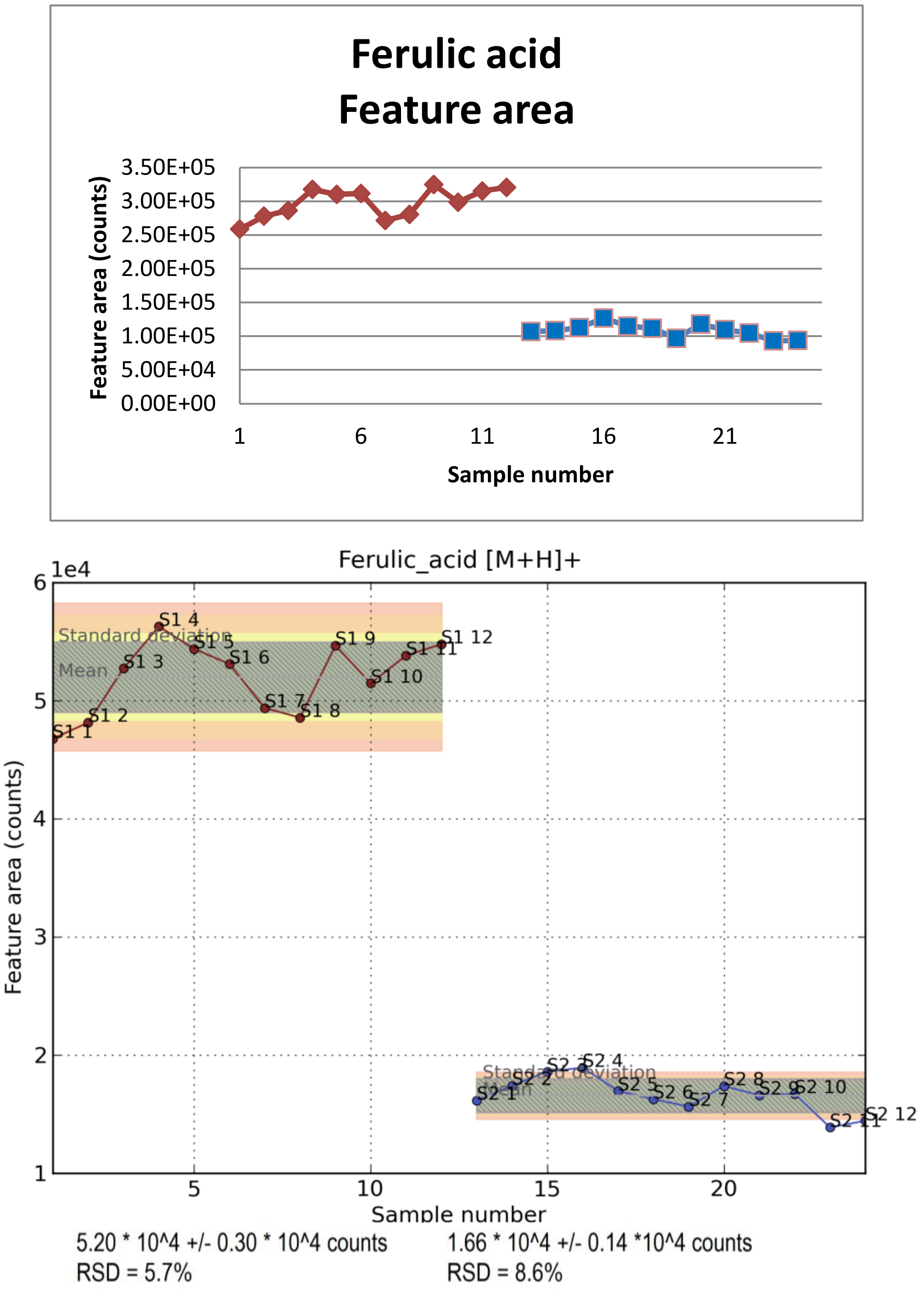

Figure S24. Plot of ferulic acid generated with MS Excel (above) based on the manual evaluation compared to the illustration generated by QCScreen (below).

		Compound		
	**Retention time**	**Ferulic acid**	Jasmonic acid	Caffeic acid
CM	**RSD (%) manual validation**	**0.20**	0.14	0.30
	**RSD (%) QCScreen**	**0.19**	0.14	0.29
Remus	**RSD (%) manual validation**	**0.30**	0.17	0.47
	**RSD (%) QCScreen**	**0.28**	0.15	0.45

Table S4. RSD for retention time of ferulic acid, jasmonic acid and caffeic acid of manual validation compared to the results generated by QCScreen.

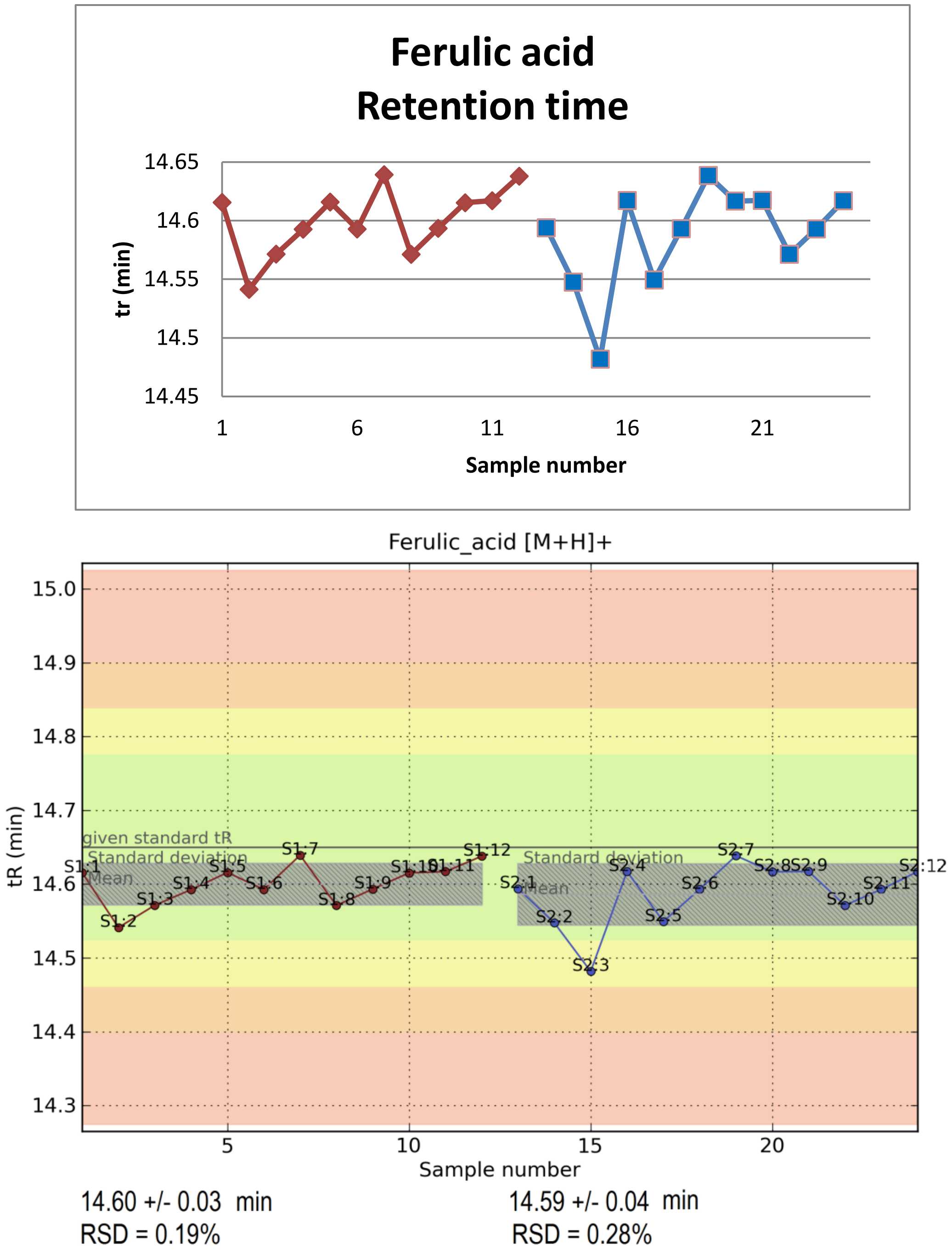

Figure S25. Plot of ferulic acid generated with MS Excel (above) based on the manual evaluation compared to the illustration generated by QCScreen (below).

		Compound		
	Mass accuracy (ppm)	Ferulic acid	Jasmonic acid	Caffeic acid
CM	RSD (%) manual validation	19	23	13
	RSD (%) QCScreen	20	20	18
Remus	RSD (%) manual validation	88	113	51
	RSD (%) QCScreen	89	107	65

Table S5. RSD for mass accuracy of ferulic acid, jasmonic acid and caffeic acid of manual validation compared to the results generated by QCScreen.

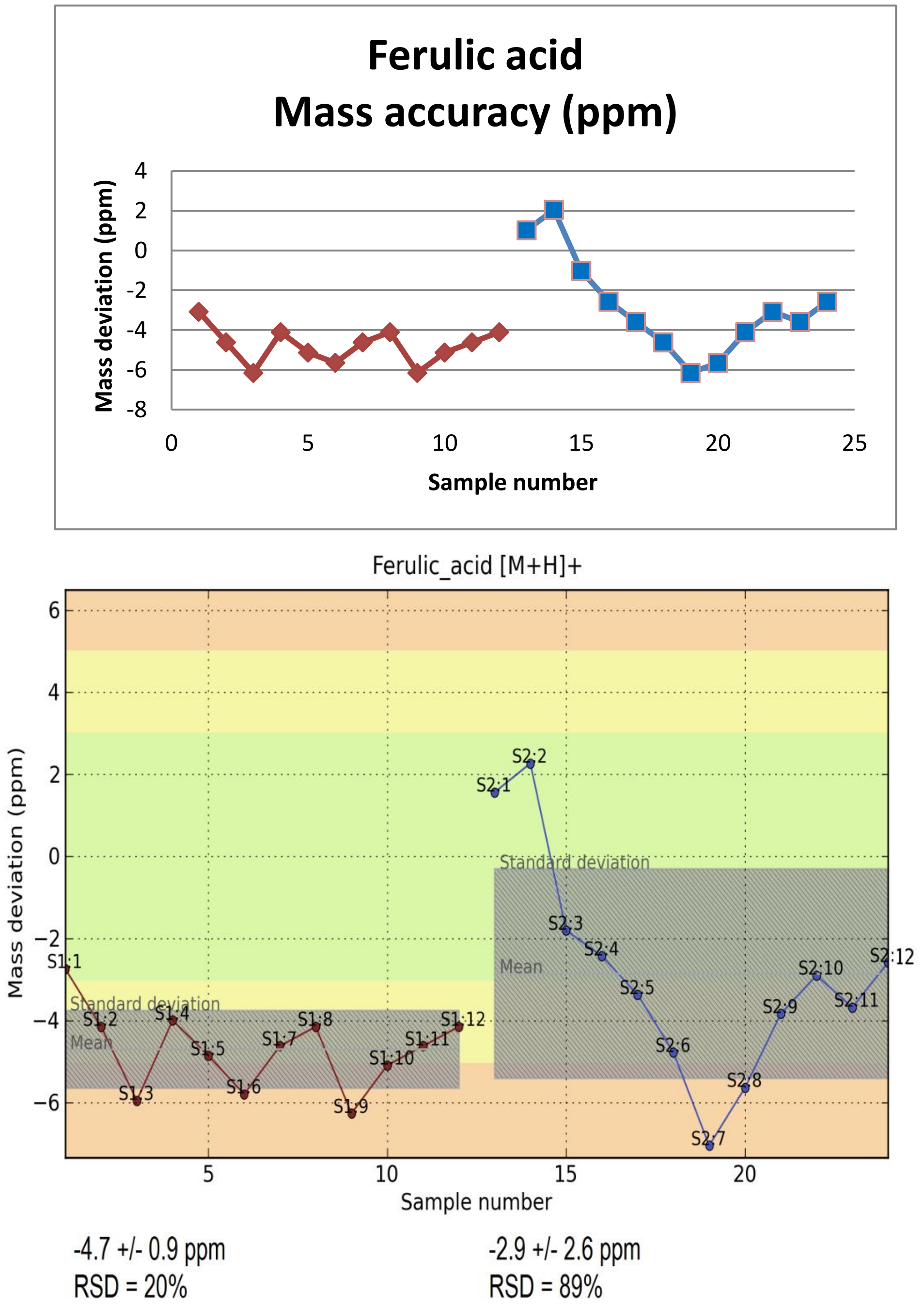

Figure S26. Plot generated with MS Excel 2007 (above) based on the manual evaluation compared to the illustration generated by QCScreen (below).

7. References

1. Warth, B*. et al*., (2014) GC-MS based targeted metabolic profiling identifies changes in the wheat metabolome following deoxynivalenol treatment. Metabolomics (2014)

2. Bueschl, C. *et al.,* (2014) A novel stable isotope labeling assisted workflow for improved untargeted LC-HRMS based metabolomics research. Metabolomics (2014) 10:754-769.
